# Supplementary material for: Real-time chirality transfer monitoring from statistically random to discrete homochiral nanotubes
Source: Nat Commun. 2022 Nov 30;13:7378. doi: 10.1038/s41467-022-34827-z (PMC9712533; doi:10.1038/s41467-022-34827-z)
Supplement: Supplementary file 1 — Supplementary Information [file 41467_2022_34827_MOESM1_ESM.pdf]

Supplementary Information

Real time chirality transfer monitoring from statistically random to discrete homochiral nanotubes

Shixin Fa[1,2,†], Tan-hao Shi[1,†], Suzu Akama[1], Keisuke Adachi[1], Keisuke Wada[1], Seigo Tanaka[1], Naoki Oyama[1], Kenichi Kato[1], Shunsuke Ohtani[1], Yuuya Nagata[3], Shigehisa Akine[4,5], Tomoki Ogoshi[1,4*]

[1]Department of Synthetic Chemistry and Biological Chemistry, Graduate School of Engineering, Kyoto University, Katsura, Nishikyo-ku, Kyoto, 615-8510, Japan
[2]School of Chemistry and Chemical Engineering, Northwestern Polytechnical University, Xi'an, Shaanxi, 710072, P.R. China
[3]WPI Institute for Chemical Reaction Design and Discovery (WPI-ICReDD), Hokkaido University, Kita 21 Nishi 10, Kita-ku, Sapporo, 060-0810, Japan
[4]WPI Nano Life Science Institute (WPI-NanoLSI), Kanazawa University, Kakuma-machi, Kanazawa, 920-1192, Japan
[5]Graduate School of Natural Science and Technology, Kanazawa University, Kakuma-machi, Kanazawa, 920-1192, Japan
[†]These authors contribute equally to this work.
[*]Correspondence to T.O.: ogoshi@sbchem.kyoto-u.ac.jp

Table of Contents

1. Synthesis……………………………………1

2. Optical resolution of 1……………………………………5

3. Chirality transfer from enantiomeric 1 to 2 via trimerization……………11

4. Chirality transfer from enantiomeric 1 to 4 via trimerization……………16

5. Chirality transfer from enantiomeric 1 to 3 via trimerization……………20

6. Mechanism of the chirality transfer process……………………21

7. Solvent effect on the chirality transfer ……………………………24

Supplementary Note 1……………………………………26

Supplementary References……………………………………27

1. Syntheses

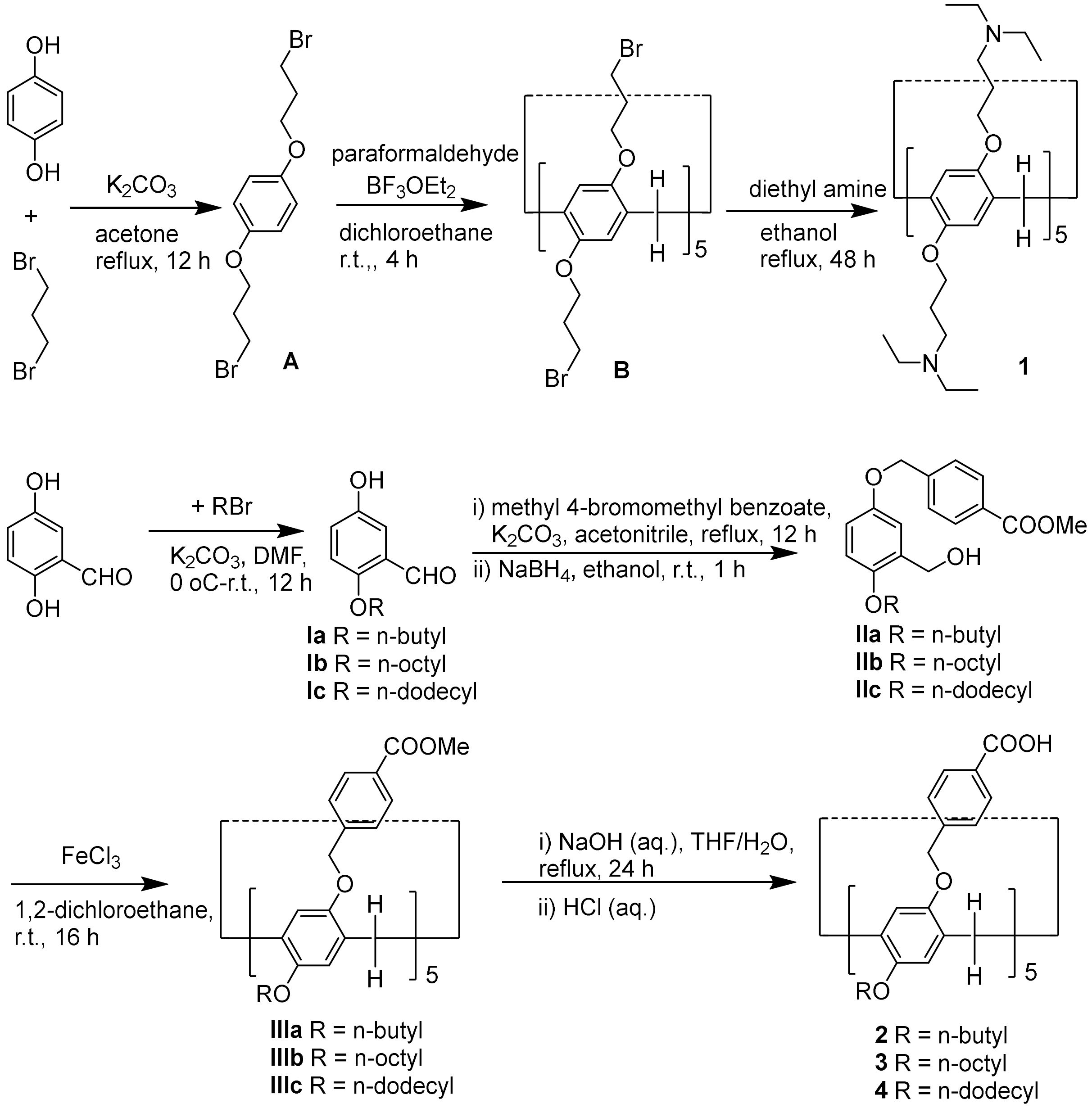

Synthesis of peraminopillar[5]arenes **1**,[1] and pentaacidic pillar[5]arenes **2**,[1] **3**[2] and **4**[1].

Compound A. Hydroquinone (2.75 g, 25 mmol) and 1,3-dibromopropane (10.2 mL, 100 mol) were dissolved in acetone (75 mL), potassium carbonate (6.90 g, 50 mmol) was added. The reaction was stirred under reflux for 12 h. The solid was filtered off after the reaction was cooling to room temperature. The solvent of the filtrate was evaporated, and the residue was chromatographed on a silica gel column using a mixture of hexane/dichloromethane (2:1, v/v) as the mobile phase. The product was obtained as white solid (5.48 g, 15.5 mmol, 62%). ^{1}H NMR (500 MHz, $CDCl_3$): δ 6.83 (s, 4H), 4.05 (t, J = 5.8 Hz, 4H), 3.60 (t, J = 5.6 Hz, 4H), 2.29 (p, J = 6.2 Hz, 4H).

Compound B. Bis(3-bromopropoxy)benzene (1.00 g, 2.84 mmol) and paraformaldehyde (170 mg, 5.68 mmol) were dissolved in dry 1,2-dichloroethane (20 mL), $BF_3 \cdot OEt_2$ (357 μL, 2.84

mmol) was added slowly at 0 °C. The reaction was stirring at room temperature for 4 h, and then quenched with water (0.5 mL). The solvent was removed under reduced pressure, and the residue was chromatographed on a silica gel column using a mixture of hexane/dichloromethane (1:1, v/v) as the mobile phase. The product was obtained as white solid (589 mg, 0.32 mmol, 57%). ^{1}H NMR (500 MHz, $CDCl_3$): *δ* 6.74 (s, 10H), 3.99 (t, *J* = 5.8 Hz, 20H), 3.76 (s, 10H), 3.51 (t, *J* = 6.5 Hz, 20H), 2.22 (p, *J* = 6.2 Hz, 20H).

Compound 1. Pillar[5]arene **B** (182 mg, 0.1 mmol)[3] was dissolved in a mixture of diethylamine (2 mL) and ethanol (10 mL). The reaction was stirred under reflux for 48 h. The solvent was then evaporated, and the residue was poured into an aqueous sodium hydroxide solution (1 M, 10 mL) and stirred. The solution was extracted with ethyl acetate (3 × 10 mL), and the organic phase was combined. After dried over anhydrous sodium sulfate, filtered and concentrated under reduced pressure, the residue was chromatographed on a silica gel column using a mixture of chloroform/methanol/triethylamine (5:1:1, v/v/v) as the mobile phase. The product was obtained as yellow oil (155 mg, 0.089 mmol, 89%). ^{1}H NMR (500 MHz, $CDCl_3$): *δ* 6.86 (s, 10H), 4.09 (dt, *J* = 8.9, 5.5 Hz, 10H), 3.85-3.76 (m, 10H), 3.75 (s, 10H), 2.77-2.64 (m, 20H), 2.56 (q, *J* = 7.2 Hz, 40H), 2.03-1.90 (m, 20H), 1.01 (t, *J* = 7.2 Hz, 60H).

General procedure of synthesis of mono-substituted dihydroxybenzaldehyde I. To a mixture of 2,5-dihydroxybenzaldehyde (10.0 g, 72.4 mmol) and 1-bromoalkanes (72.4 mmol) in dry DMF (100 mL) was added potassium carbonate (20.0 g, 145 mmol). The mixture was stirred at room temperature for 24 h. The solid was filtered off, and the filtrate was concentrated under reduce pressure. The residue was dissolved in ethyl acetate (100 mL) and washed with water (3 × 25 mL) and brine (25 mL), dried over anhydrous sodium sulfate and filtered. After removal of the solvent, the residue was chromatographed on a silica gel column using a mixture of *n*-hexane and ethyl acetate (from 10:1 to 5:1, v/v) as the mobile phase.

Compound Ia. Prepared from 1-bromobutane (7.8 mL, 72.4 mmol) and obtained as yellow solid (5.54 g, 28.6 mmol, 39%). ^{1}H NMR (400 MHz, $CDCl_3$): *δ* 10.41 (s, 1H), 7.37 (d, *J* = 3.1 Hz, 1H), 7.10 (dd, *J* = 9.0, 3.2 Hz, 1H), 6.88 (d, *J* = 8.9 Hz, 1H), 6.45 (s, 1H), 4.01 (t, *J* = 6.4 Hz, 2H), 1.84-1.73 (m, 2H), 1.56-1.42 (m, 2H), 1.01-0.93 (m, 3H).

Compound Ib. Prepared from 1-bromooctane (12.6 mL, 72.4 mmol) and obtained as yellow solid (8.17 g, 32.6 mmol, 45%). ^{1}H NMR (500 MHz, $CDCl_3$): *δ* 10.44 (s, 1H), 7.37-7.33 (m, 1H), 7.13-7.06 (m, 1H), 6.90 (d, *J* = 9.0 Hz, 1H), 4.02 (t, *J* = 6.5 Hz, 2H), 1.86-1.77 (m, 2H), 1.51-1.42 (m, 2H), 1.39-1.23 (m, 16H), 0.92-0.86 (m, 3H).

Compound Ic. Prepared from 1-bromododecane (17.4 mL, 72.4 mmol) and obtained as yellow solid (5.54 g, 30.0 mmol, 41%). ^{1}H NMR (400 MHz, $CDCl_3$): *δ* 10.43 (s, 1H), 7.33 (d, *J* = 3.2 Hz, 1H), 7.08 (dd, *J* = 8.9, 3.2 Hz, 1H), 6.88 (d, *J* = 8.9 Hz, 1H), 5.59 (s, 1H), 4.01 (t, *J* = 6.4 Hz, 2H), 1.85-1.75 (m, 2H), 1.51-1.39 (m, 2H), 1.38-1.20 (m, 16H), 0.90-0.82 (m, 3H).

General procedure of synthesis of di-substituted dihydroxybenzyl alcohol II. To a mixture of **I** (10 mmol) and methyl 4-(bromomethyl) benzoate (2.75 g, 12 mmol) in acetonitrile (100 mL) was added potassium carbonate (1.66 g, 12 mmol). The reaction mixture was stirred under reflux for 12 h. The solid was filtered off, and the filtrate was concentrated under reduced pressure. The residue was suspended in ethanol (30 mL) and cooled down to 0 °C with an ice

bath. Sodium borohydride (460 mg, 12 mmol) was then carefully added. The ice bath was removed, and the mixture was stirred at room temperature for 1 h. Saturated NH_4Cl (aq. 50 mL) was added carefully to quench the reaction. The organic solvent was removed under reduced pressure, and the residue was extracted with dichloromethane (3 × 50 mL). The combined organic phase was washed with water (50 mL) and brine (50 mL), dried over anhydrous sodium sulfate and filtered. After removal of the solvent, the residue was chromatographed on a silica gel column using a mixture of dichloromethane and ethyl acetate (from 60:0 to 60:1, v/v) as the mobile phase to get the products as white solid.

Compound IIa. Prepared from **Ia** (1.94 g, 12 mmol) and 3.18 g (92%) of product was obtained. ^{1}H NMR (400 MHz, $CDCl_3$): δ 8.04 (d, J = 8.6 Hz, 2H), 7.49 (d, J = 8.0 Hz, 2H), 6.95 (d, J = 2.8 Hz, 1H), 6.85-6.74 (m, 2H), 5.08 (s, 2H), 4.66 (t, J = 2.8 Hz, 2H), 3.96 (t, J = 6.4 Hz, 2H), 3.92 (s, 3H), 1.83-1.71 (m, 2H), 1.56-1.42 (m, 2H), 0.98 (t, J = 7.4 Hz, 3H).

Compound IIb. Prepared from **Ib** (2.50, 12 mmol) and 3.64 g (91%) of product was obtained. ^{1}H NMR (400 MHz, $CDCl_3$): δ 8.05 (d, J = 8.3 Hz, 2H), 7.49 (d, J = 8.0 Hz, 2H), 6.95 (d, J = 2.3 Hz, 1H), 6.82 (dd, J = 8.8, 2.8 Hz, 1H), 6.78 (d, J = 8.9 Hz, 1H), 5.08 (s, 2H), 4.66 (d, J = 6.6 Hz, 2H), 3.96 (t, J = 6.1 Hz, 2H) 3.92 (s, 3H), 2.45-2.36 (m, 1H), 1.83-1.73 (m, 2H), 1.49-1.41 (m, 2H), 1.39-1.22 (m, 8H), 0.93-0.85 (m, 3H).

Compound IIc. Prepared from **Ic** (3.07 mL, 12 mmol) and 4.12 g (90%) of product was obtained. ^{1}H NMR (400 MHz, $CDCl_3$): δ 8.04 (d, J = 8.3 Hz, 2H), 7.49 (d, J = 8.1 Hz, 2H), 6.95 (d, J = 2.8 Hz, 1H), 6.81 (dd, J = 8.8, 2.9 Hz, 1H), 6.77 (d, J = 8.8 Hz, 1H), 5.08 (s, 2H), 4.66 (d, J = 6.4 Hz, 2H), 3.95 (t, J = 6.5 Hz, 2H) 3.92 (s, 3H), 2.48-2.39 (m, 1H), 1.84-1.72 (m, 2H), 1.48-1.38 (m, 2H), 1.39-1.20 (m, 16H) 0.91-0.85 (m, 3H).

General procedure of synthesis of rim-different pillar[5]arene ester III. To a stirred solution of **II** (2 mmol) in 1,2-dichloroethane (200 mL) was added anhydrous iron (III) chloride (33.0 mg, 0.2 mmol). The mixture was stirred at room temperature for 16 h before methanol (50 mL) was added to quench the reaction. The solution was washed by water (100 mL) and brine (100 mL), dried over anhydrous sodium sulfate, filtered and concentrated under reduced pressure. The residue was chromatographed on a silica gel column using a mixture of *n*-hexane and acetone (from 10:1 to 5:1, v/v) as the mobile phase to obtain crude products as pale yellow solid. The products were then recrystallized from ether/*n*-hexane, and white solid was obtained.

Compound IIIa. Prepared from **IIa** (690 mg, 2 mmol) and 111 mg (17%) of product was obtained. ^{1}H NMR (400 MHz, $CDCl_3$): δ 7.98 (d, J = 8.3 Hz, 10H), 7.34 (d, J = 8.3 Hz, 10H), 6.88 (s, 5H), 6.78 (s, 5H), 4.52 (s, 10H), 3.93-3.85 (m, 25H), 3.83 (s, 10H), 1.83-1.73 (m, 10H), 1.59-1.49 (m, 10H), 0.98 (t, J = 7.4 Hz, 15H).

Compound IIIb. Prepared from **IIb** (801 mg, 2 mmol) and 98 mg (13%) of product was obtained. ^{1}H NMR (400 MHz, $CDCl_3$): δ 7.96 (d, J = 8.2 Hz, 10H), 7.33 (d, J = 8.2 Hz, 10H), 6.86 (s, 5H), 6.79 (s, 5H), 4.57-4.44 (m, 5H), 3.94-3.74 (m, 40H), 1.93-1.70 (m, 10H), 1.54-1.46 (m, 10H), 1.36-1.29 (m, 10H), 1.27-1.21 (m, 30H), 0.84-0.80 (m, 15H).

Compound IIIc. Prepared from **IIc** (913 mg, 2 mmol) and 166 mg (19%) of product was obtained. ^{1}H NMR (400 MHz, $CDCl_3$): δ 7.99 (d, J = 8.3 Hz, 10H), 7.31 (d, J = 8.4 Hz, 10H), 6.90 (s, 5H), 6.84 (s, 5H), 4.68-4.27 (m, 10H), 4.07-3.71 (m, 35H), 1.94-1.73 (m, 10H), 1.59-1.46 (m, 10H), 1.37 (p, J = 7.2 Hz, 10H), 1.27 (p, J = 6.9 Hz, 10H), 1.21-0.84 (m, 60H), 0.78 (t, J = 7.2 Hz, 15H).

General procedure of synthesis of rim-different pillar[5]arene acid. To the solution of **III** (0.03 mmol) in THF (2.5 mL), sodium hydroxide (36 mg, 0.9 mmol) in water (2.5 mL) was added dropwise. The mixture was refluxed for 24 h. After the organic solvent was removed under reduced pressure, the solution was treated with aqueous HCl solution (1 M) until pH 1. The precipitate was collected by filtration, and washed with water (200 mL). After drying in vacuum, the product was obtained.

Compound 2. Prepared from **IIIa** (49 mg, 0.03 mmol) and 42 mg (90%) of product was obtained as pale yellow solid. ^{1}H NMR (500 MHz, DMSO-d_6): δ 7.93 (d, J = 8.3 Hz, 10H), 7.46 (d, J = 8.4 Hz, 10H), 6.90 (s, 5H), 6.78 (s, 5H), 4.69 (s, 10H), 3.85 (t, J = 6.6 Hz, 10H), 3.73, (s, 10H), 1.75-1.68 (m, 10H), 1.52-1.41 (m, 10H), 0.92 (t, J = 7.4 Hz, 15H).

Compound 3. Prepared from **IIIb** (57 mg, 0.03 mmol) and 43 mg (78%) obtained as yellow solid. ^{1}H NMR (400 MHz, $CDCl_3$): δ 8.00 (d, J = 7.8 Hz, 10H), 7.29 (d, J = 7.9 Hz, 10H), 6.89 (s, 5H), 6.87 (s, 5H), 4.77 (d, J = 13.7 Hz, 5H), 4.49 (d, J = 13.8 Hz, 5H), 4.04-3.79 (m, 20H), 1.94-1.75 (m, 10H), 1.62-1.49 (m, 10H), 1.42-1.35 (m, 10H), 1.34-1.25 (m, 10H), 1.24-1.09 (m, 20H), 0.89-0.77 (t, 15H).

Compound 4. Prepared from **IIIc** (66 mg, 0.03 mmol) and 58 mg (91%) obtained as pale yellow solid. ^{1}H NMR (500 MHz, DMSO-d_6, 393.15 K): δ 7.92 (d, J = 7.9 Hz, 10H), 7.42 (d, J = 8.0 Hz, 10H), 6.88 (s, 5H), 6.80 (s, 5H), 4.66 (s, 10H), 3.83 (t, J = 6.6 Hz, 10H), 3.73 (s, 10H), 1.81-1.73 (m, 10H), 1.54-1.44 (m, 10H), 1.41-1.10 (m, 80H), 0.81 (t, J = 6.7 Hz, 15H).

2. Optical resolution of 1

2.1 Spectra of racemic 1 and the two enantiomers

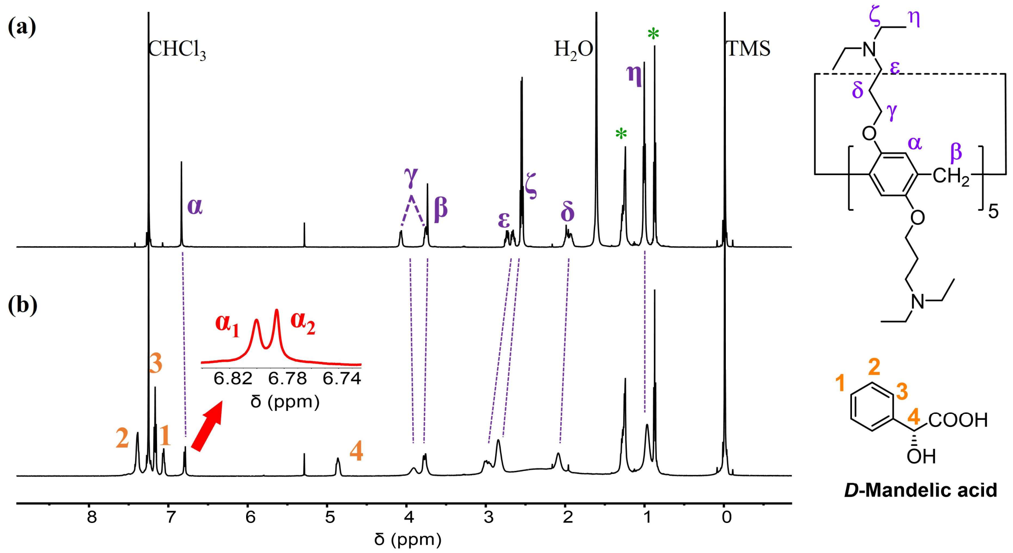

Supplementary Fig. 1 Full ^{1}H NMR spectra (600 MHz, 25 °C, $CDCl_3$) of (a) racemic **1** and (b) the mixture of racemic **1** with 10 equiv. of *D*-mandelic acid. The peaks marked with asterisk are ascribed to *n*-hexane.

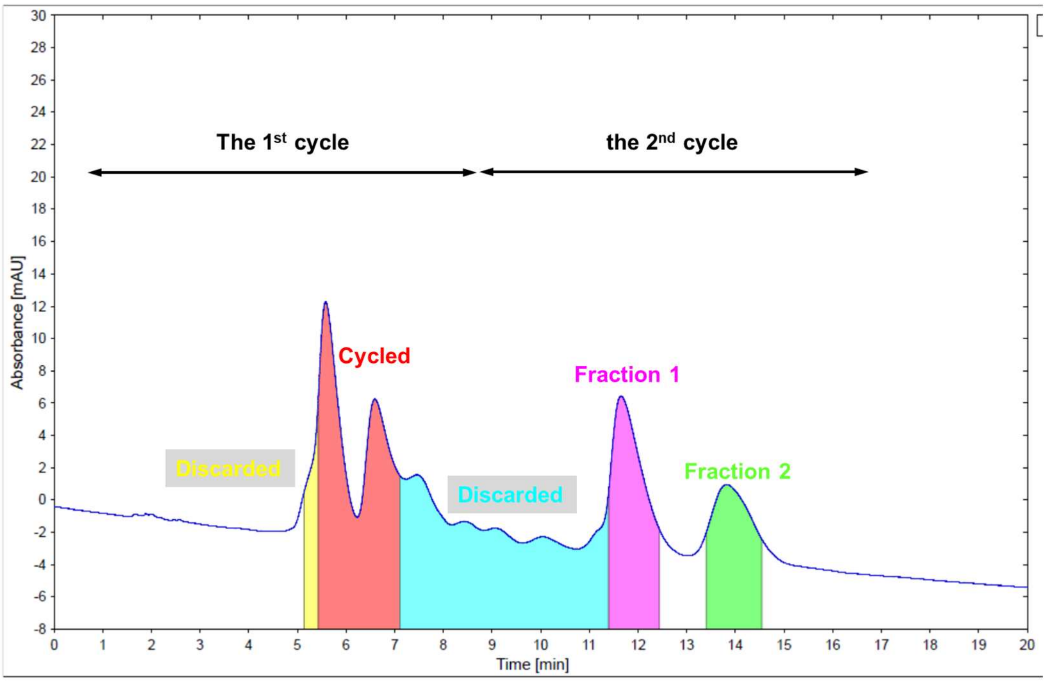

Supplementary Fig. 2 Optical resolution of **1**. The HPLC trace (*n*-hexane/ EtOH/ ethylenediamine, 92.5/ 7.5/ 0.1, v/v/v, 25 °C) was collected on a JAI LaboACE LC-5060 HPLC equipped with a DAICEL CHIRALPAK® IE (ϕ = 10 mm, l = 250 mm) column.

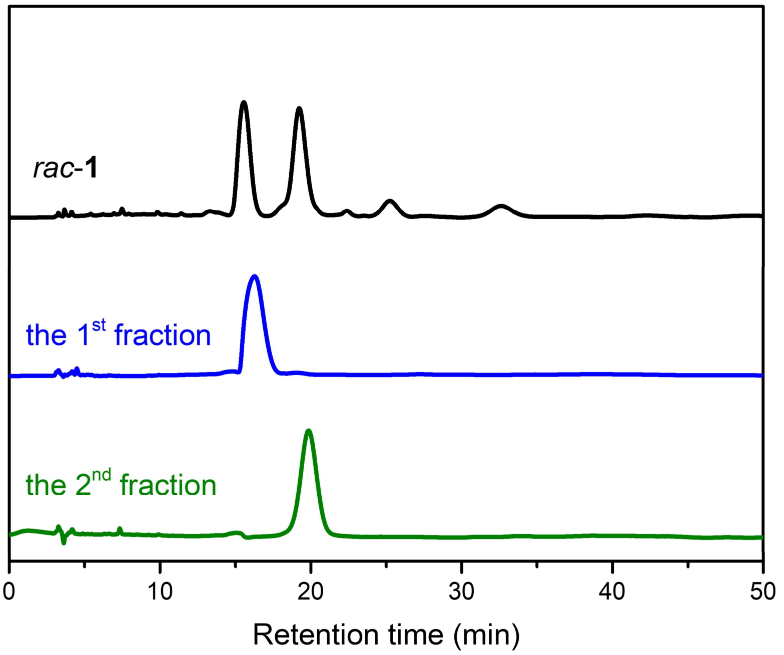

Supplementary Fig. 3 Chiral HPLC traces (*n*-hexane/ EtOH/ ethylenediamine, 92.5/ 7.5/ 0.1, v/v/v, 25 °C) of *rac*-**1** and the re-injected first (*pR*-**1**) and second (*pS*-**1**) fractions. Column: DAICEL CHIRALPAK® IE (ϕ = 4.6 mm, l = 250 mm) column.

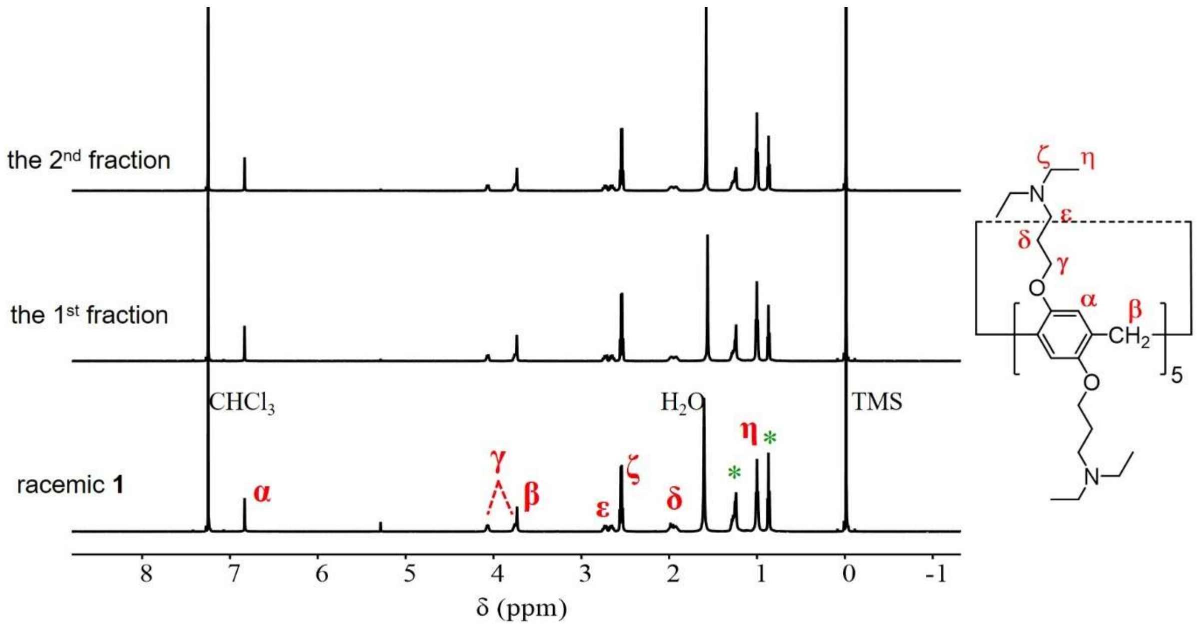

Supplementary Fig. 4 Full ^{1}H NMR spectra (600 MHz, 25 °C, $CDCl_3$) of racemic **1** and the two fractions of chiral resolution (0.5 mM). The peaks marked with asterisk are ascribed to *n*-hexane.

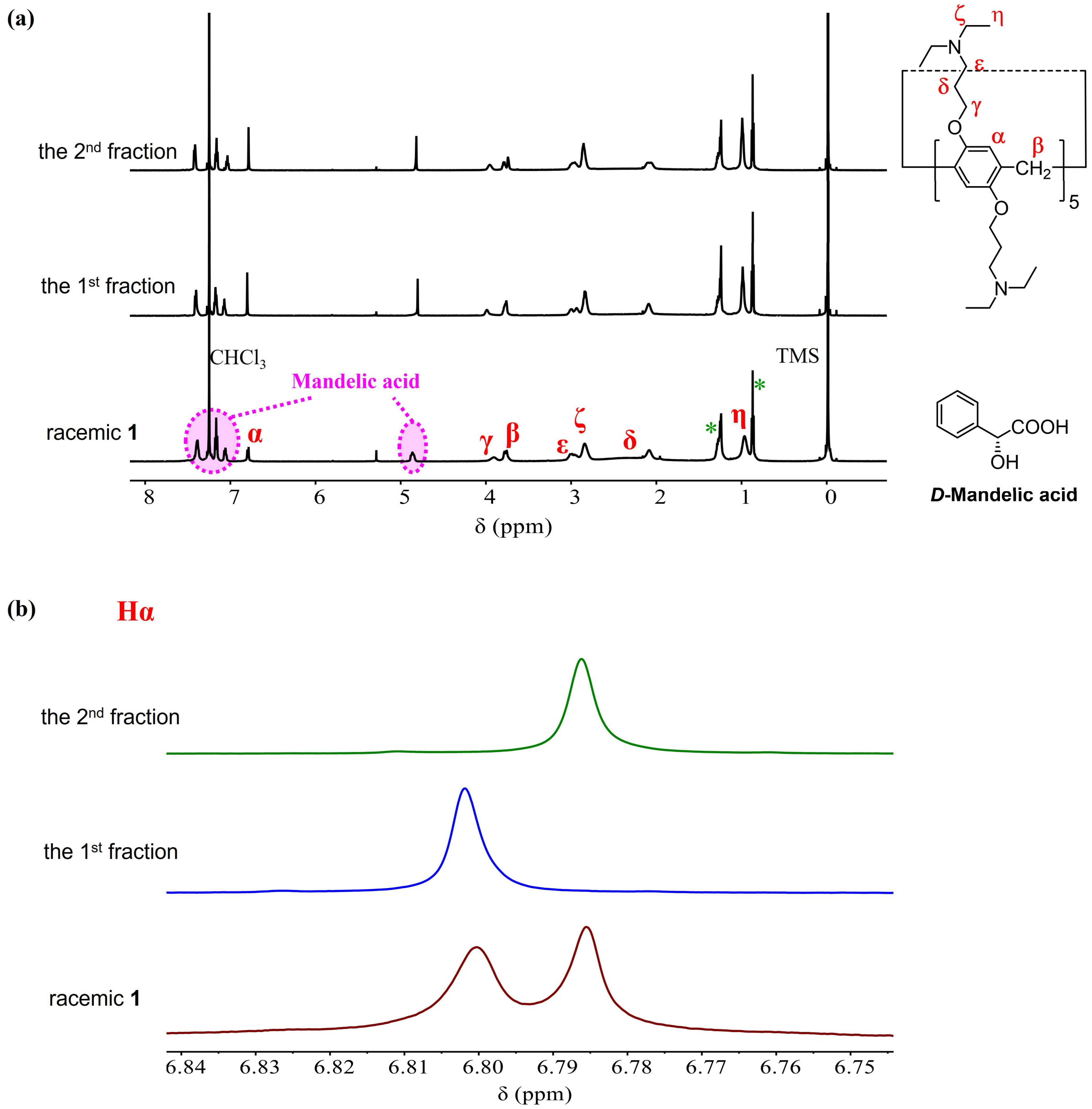

Supplementary Fig. 5 (a) Full and (b) partial ^{1}H NMR spectra (600 MHz, 25 °C, $CDCl_3$) of racemic **1** and the two fractions (0.5 mM) with 10 equiv. of *D*-mandelic acid. The peaks marked with asterisk are ascribed to *n*-hexane.

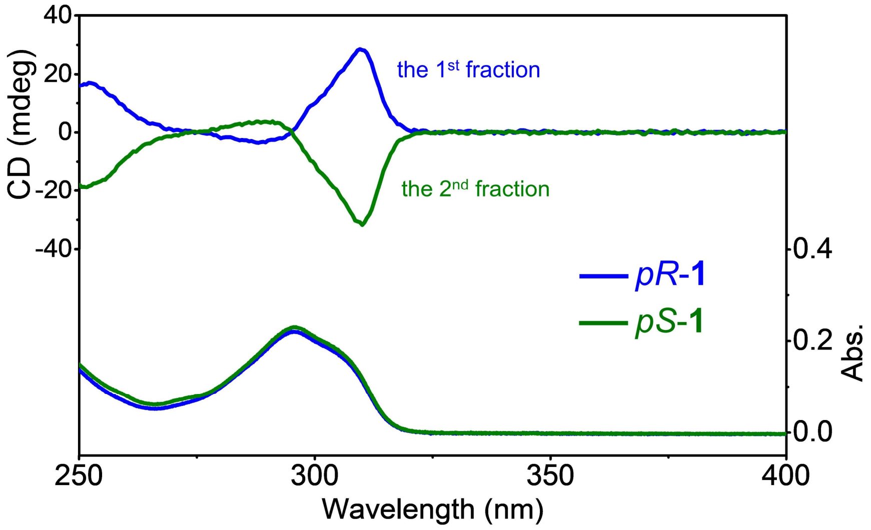

Supplementary Fig. 6 UV–Vis and CD spectra (0.05 mM, chloroform, 25 °C) of *pR*-**1** and *pS*-**1**.

2.2 Stability of enantiomeric 1

The stability of the isolated enantiomeric **1** was initially determined by Eyring equation of the thermal racemization of *pS*-**1** (Supplementary Fig. 7). The solutions of *pS*-**1** in chloroform (0.05 mM) in a 2 mm quartz cuvette were heated to 55 °C, 50 °C, 45 °C and 40 °C, respectively. The CD intensity of the samples at 310 nm was recorded in 3 h. The thermodynamic parameters such as $\Delta H^{\neq}$, $\Delta S^{\neq}$ were determined from the slope and intercept by plotting $\ln(hk/k_{\mathrm{B}}T)$ *vs* $1/T$ by using the linear Eyring plots[4]:

$$\ln\frac{hk}{k_{\mathrm{B}}T} = -\frac{\Delta H^{\neq}}{R}\frac{1}{T} + \frac{\Delta S^{\neq}}{R}$$

where h, k_{B} and R are Planck constant, Boltzmann constant and molar gas constant, respectively. k is the reaction rate constant. By using the above equation, we speculated the thermal racemization of *pS*-**1** as a pseudo-first order reaction. $\Delta G^{\neq}$ was obtained according to the following equation:

$$\Delta G^{\neq} = \Delta H^{\neq} - T\Delta S^{\neq}$$

Considering that the structure of **1** is flexible due to the methylene bridges, the phenyl units of planar chiral **1** can swing in the solution. Higher temperature makes the swing more violent, which reduces the CD intensity even more. That is to say, the decrease of the signal during the heating process comes from two factors, one is the racemization, and the other is the intensified swing of the molecule. Therefore, it may be not accurate to determine the stability of enantiomeric **1** via thermal racemization.

We recorded the CD spectra of *pS*-**1** at 25 °C in 4 weeks (Supplementary Fig. 8). The observations suggested high stability of *pS*-**1**.

However, too high a temperature will rapidly racemize *pS*-**1**. The CD change of *pS*-**1** was also monitored at 100 °C. Taking into account the factors of solubility and boiling point, we used tetrachloroethane as the solvent. The CD signal of *pS*-**1** at 100 °C in tetrachloroethane

completely disappeared within 3 h, and no signal was observed even when it cooled back to room temperature (Supplementary Fig. 9).

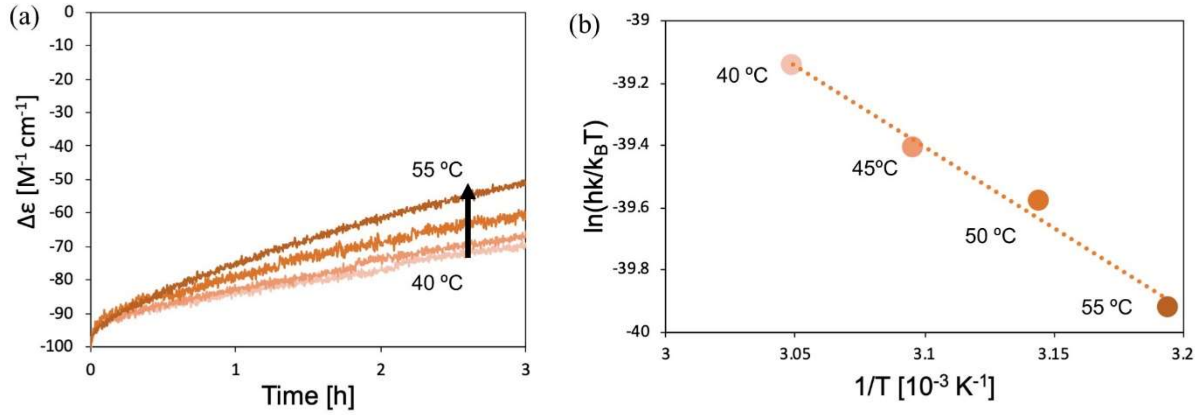

Supplementary Fig. 7 Kinetic study for the thermal racemization of *pS*-**1**. (a) Time dependent CD spectra of *pS*-**1** (0.05 mM, chloroform) at 310 nm at various temperatures. (b) Eyring plots from the CD changes of *pS*-**1**. The $\Delta H^{\neq}$ and $\Delta S^{\neq}$ were determined to be 43.0 kJ/mol, and −194 J/mol/K, respectively. Therefore, the Gibbs energy of activation at 25 °C was determined to be $\Delta G^{\ddagger}_{25°C}$ = 101 kJ/mol.

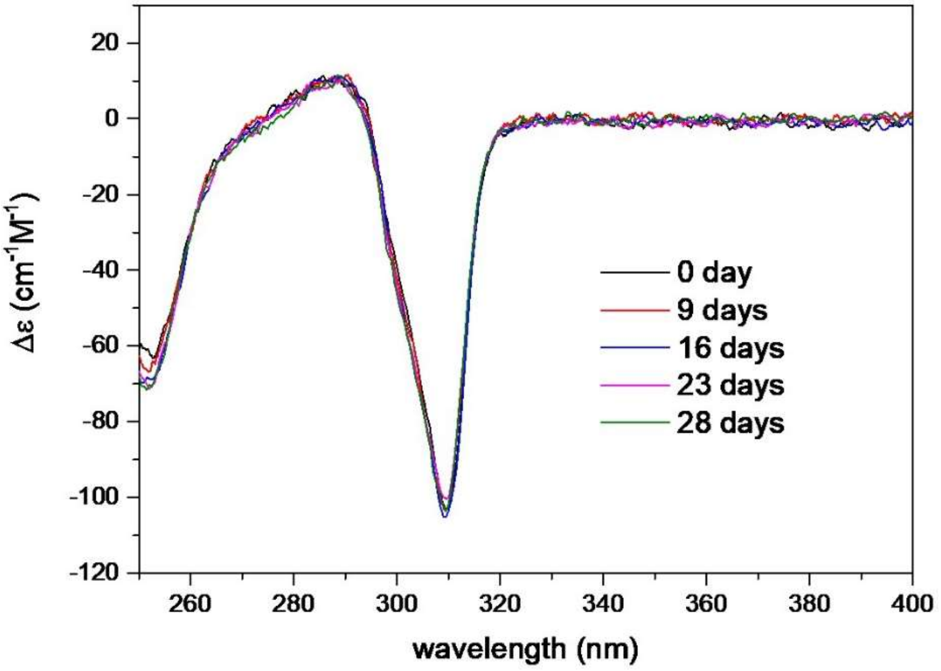

Supplementary Fig. 8 Full CD spectra of *pS*-**1** (0.05 mM, chloroform, 25 °C) recorded within 4 weeks.

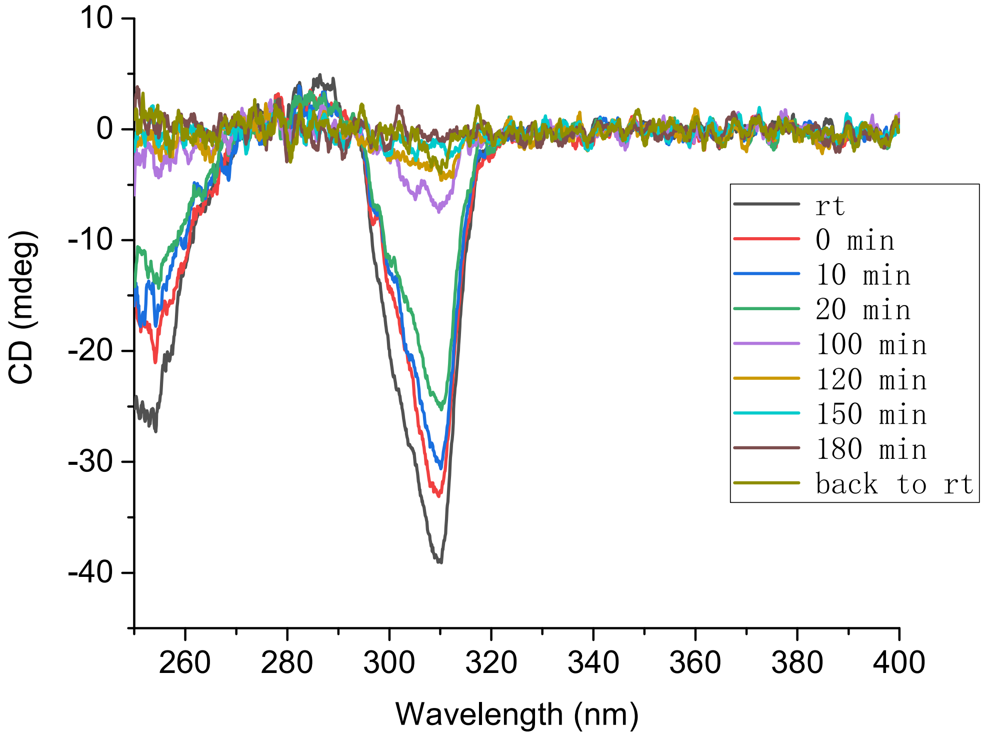

Supplementary Fig. 9 Full CD spectra of *pS*-**1** (0.05 mM, tetrachloroethane, 100 °C).

3. Chirality transfer from enantiomeric 1 to 2 via trimerization

3.1 Preparation of the trimer

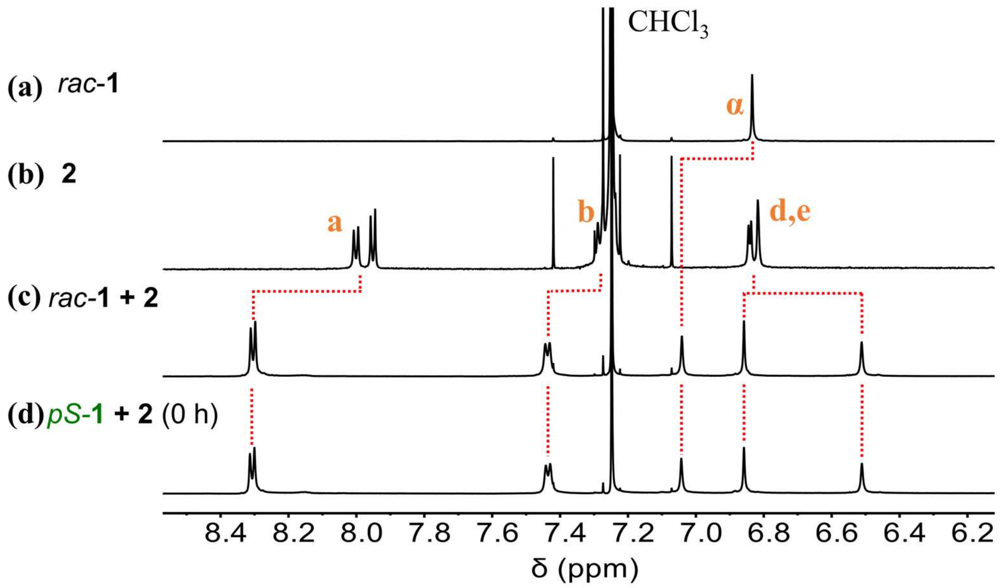

Supplementary Fig. 10 Partial ^{1}H NMR spectra (600 MHz, $CDCl_3$, 25 °C) of (a) racemic **1** (0.5 mM), (b) **2** (1.0 mM), (c) the mixture of racemic **1** (0.5 mM) and **2** (1.0 mM), and (d) the mixture of *pS*-**1** (0.5 mM) and **2** (1.0 mM). The sample in (d) was measured as prepared, whose spectrum was exactly same as that in (c) and appeared to be no change after long time. This suggested that: (1) The same assemblies were formed in (c) and (d); (2) the assembly in (d) was quickly formed after mixing; and (3) only one kind of assembly was formed in (d).

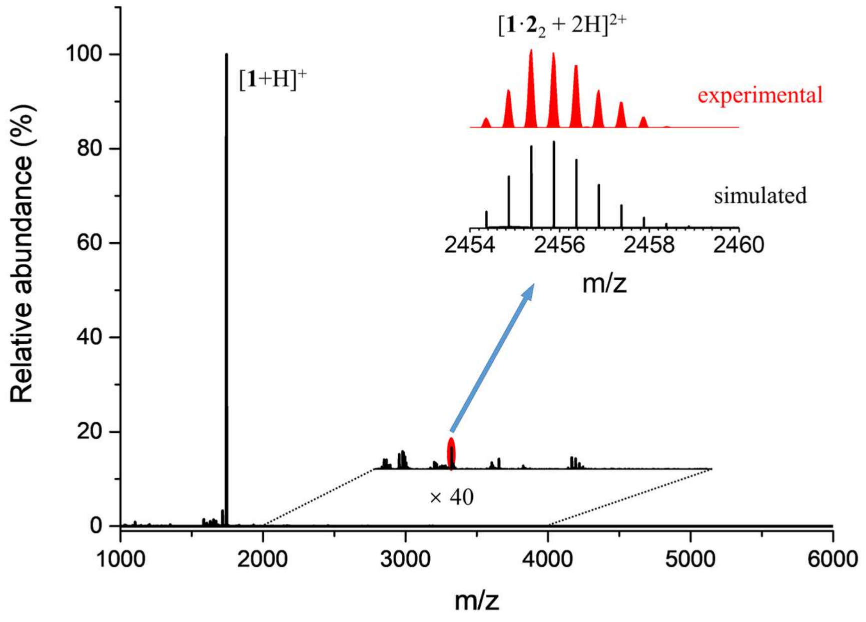

Supplementary Fig. 11 High resolution ESI-mass spectra of the mixture of *pS*-**1** and **2** (1:2).

3.2 The evidence on the "swing effect" of the monomer and trimer

As discussed in the main text, the CD intensity of the mixture of *pS*-**1** and **2** in a 1:2 molar ratio was about 5 times that of *pS*-**1**, which made the efficiency of chirality transfer appear to be over 100%. To explain this observation, we speculated that the additional increase in CD intensity came from structural fixing caused by trimerization. Therefore, we proposed the "swing effect" of pillar[n]arenes on the CD intensity:

Even though flip of the units of a pillar[n]arene with bulky substituents is inhibited due to steric hindrance, the non-rigid methylene bridges may allow the units to "swing" while maintaining the planar chirality of the pillar[n]arene. Such a "swing effect" makes the molecule not always perfectly pillar-shaped in solution. Thus, the "apparent CD intensity" may not be maximized. Guest inclusion and self-assembly can stop the molecule from swinging, which helps to increase the CD intensity (Supplementary Fig. 12).

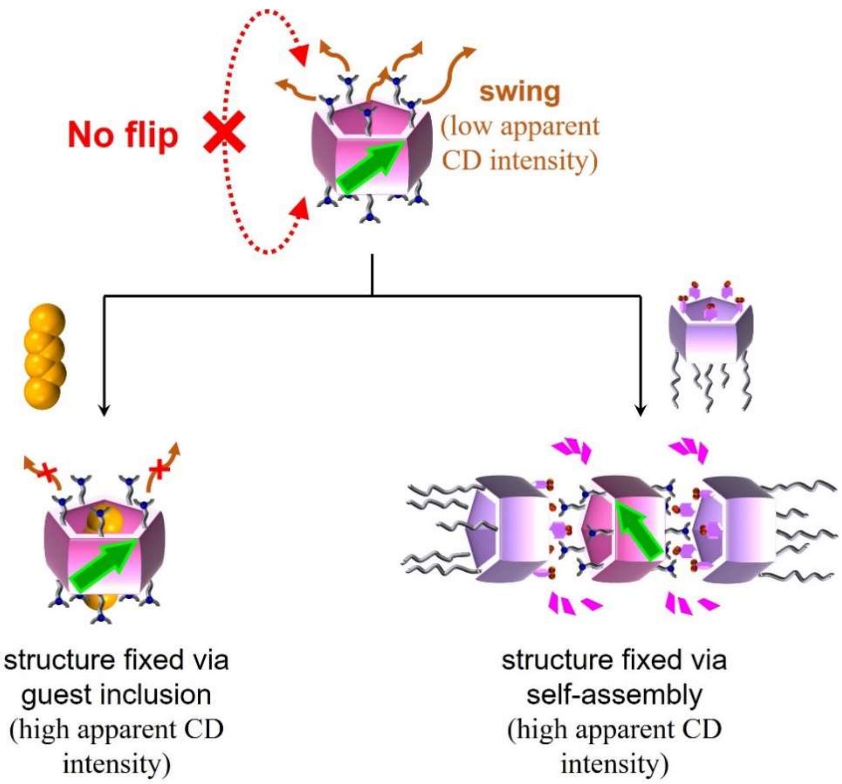

Supplementary Fig. 12 Schematic illustration of the "swing effect" of pillar[n]arenes and the structural fixation to suppress the "swing effect".

The "swing effect" was confirmed by the experiments on host–guest complexation and self-assembly. Supplementary Fig. 13a shows the fivefold CD increase as trimer formation. Upon addition of 2 equiv. of 1,4-dibromobutane (DBB), a known guest of pillar[5]arenes, the CD intensity of *pS*-**1** increased by 50% (Supplementary Fig. 13b), clearly suggesting that the structural fixation of *pS*-**1** through host–guest complexation suppressed the swing of pillar[5]arene units. Similarly, addition of 6 equiv. of DBB in the mixture of *pS*-**1** and **2** (1:2) caused the CD intensity to increase slightly (Supplementary Fig. 13c), which suggested that

trimerization of *pS*-**1** and **2** had fixed the structure of the pillar[5]arenes successfully, the addition of DBB showed only a very limited effect on the further fixation of the structure. Therefore, the CD intensity of the trimer was three times that of guest-included *pS*-**1** (Supplementary Fig. 13d), indicating a perfect chirality transfer (almost 100%) from *pS*-**1** to **2**.

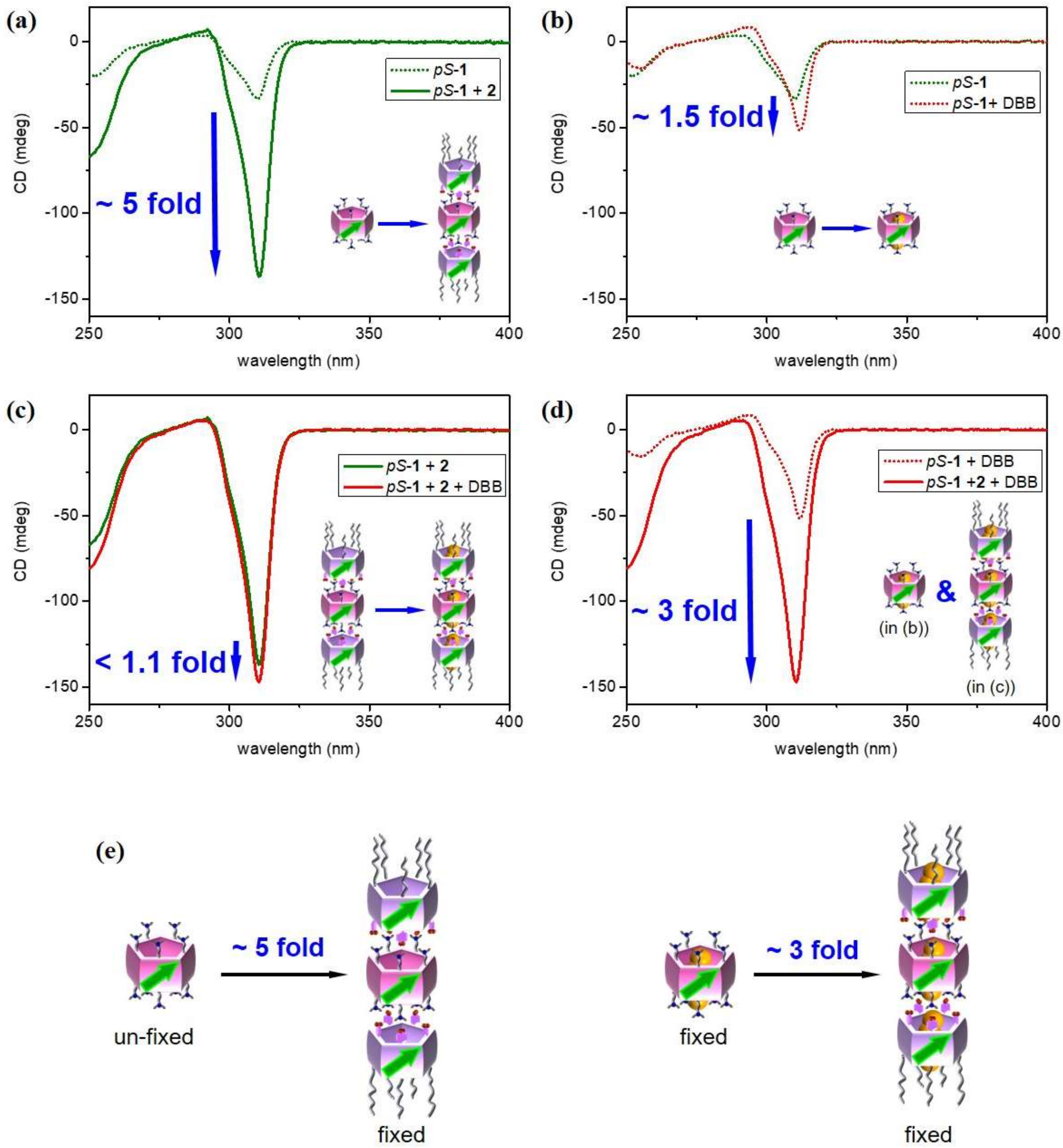

Supplementary Fig. 13 The swing effect on the CD intensity of *pS*-**1** and the suppression of the unit swing. (a) CD change as trimerization. (b) CD change of *pS*-**1** upon addition of DBB. (c) CD change of the trimer upon addition of DBB. (d) Comparison of the CD intensities of *pS*-**1** with fixed structure and the corresponding trimer formed with **2**. In all cases, the concentrations of **1** and **2** were 0.05 mM and 0.1 mM, respectively. (e) Schematic illustration of CD increase influenced by the "swing effect".

3.3 Fast chirality transfer from enantiomeric 1 to 2

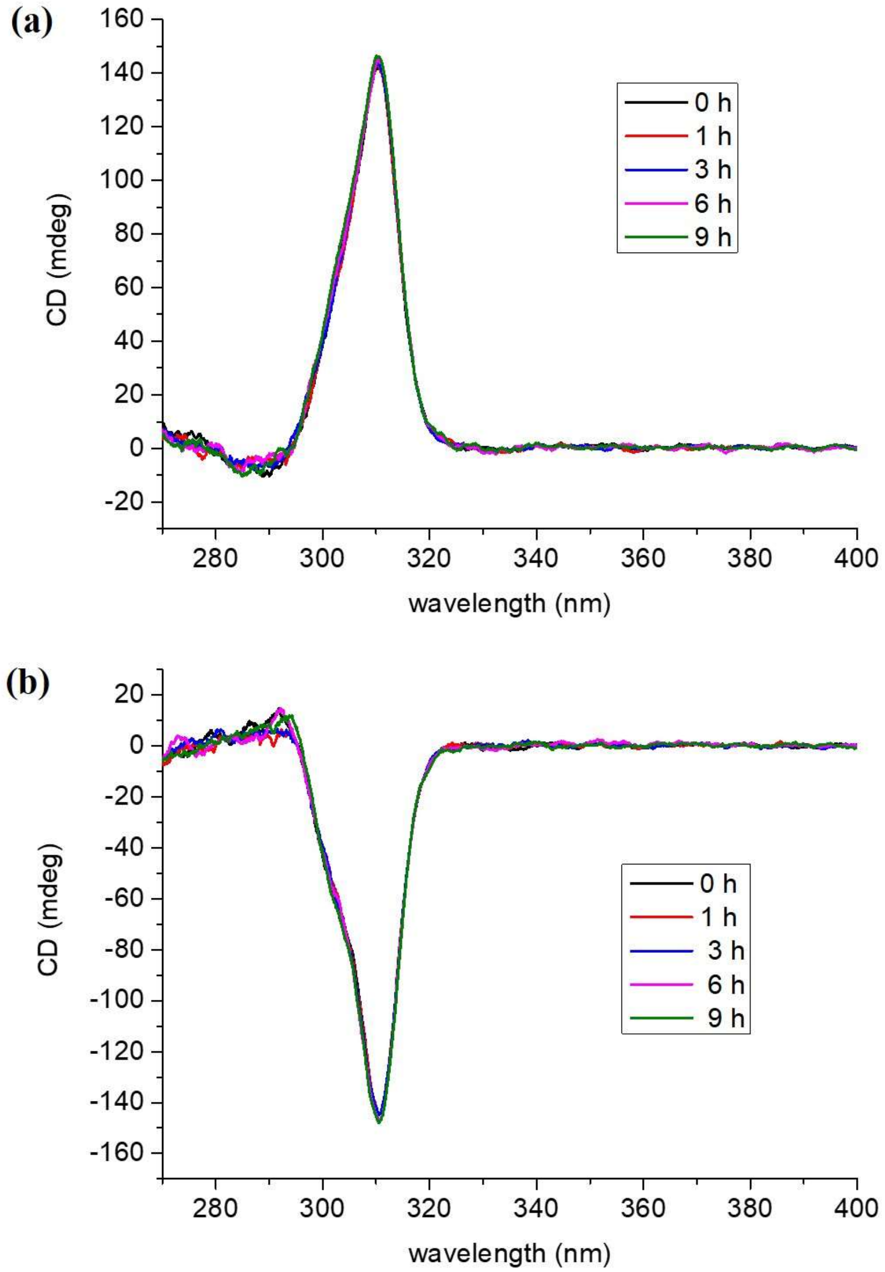

Supplementary Fig. 14 CD spectra of (a) the mixture of *pR*-**1** (0.5 mM) with **2** (1.0 mM) in chloroform upon heating at 50 °C and (b) the mixture of *pS*-**1** (0.5 mM) with **2** (1.0 mM) in chloroform upon heating at 50 °C. The CD intensity hardly changed upon heating within a period of 9 h, suggesting that the chirality transfer from enantiomeric **1** to **2** was completed immediately after mixing.

3.4 Computational data of the heterochiral and homochiral trimers

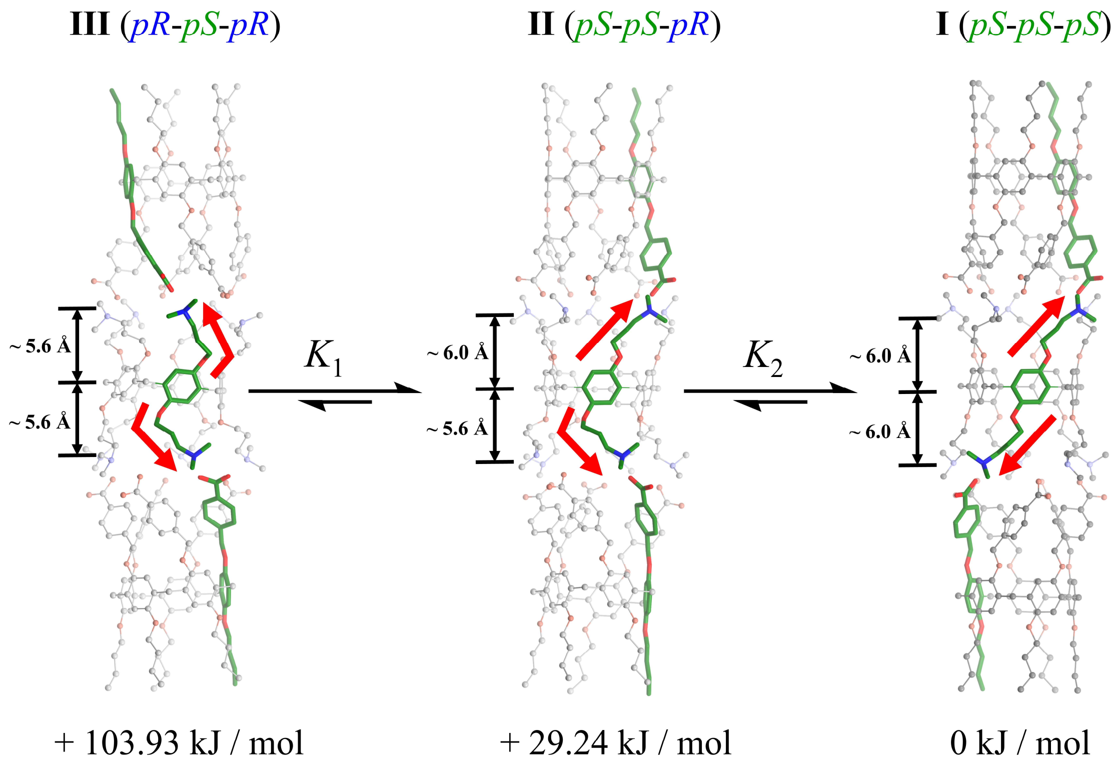

Supplementary Fig. 15 Optimized structures and relative energies of the three possible trimers of the mixture of **2** with *pS*-**1** in chloroform (ORCA 4.2.3 with the semiempirical GFN2-xTB method and the SMD solvation model).[5-7] To simplify the system, the ten 3-(diethylamino)propoxy groups in **1** are replaced by 3-(dimethylamino)propoxy groups. It is clear that the substitutes of *pS*-**1** show a turn when it interacts with *pR*-**2**, so as to offset the error caused by the mismatch of planar chirality. This results in a reduction in the effective length of the substituents of *pS*-**1**, which increases the steric hindrance between the two pillar[5]arenes when they interact with each other through ionic interactions. Therefore, the strength of the ionic interaction between *pS*-**1** and *pR*-**2** is naturally smaller than that between *pS*-**1** and *pS*-**2**, although both of them are ten pairs of ionic interaction between tertiary amines and benzoic acids. Based on the relative energies between the three trimers, the equilibrium constants K_1 and K_2 shown above can be determined using the equation: $\Delta G = -\mathrm{RTln}K$. K_1 and K_2 were 1.4×10^{13} and 1.3×10^{5}, respectively.

Supplementary Table 1. Theoretical calculation of the relative energy of the three types of trimer.

Complex	Energy (hartree)	Energy (kJ/mol)	Relative energy (kJ/mol)
**I** (*pS*-*pS*-*pS*)	−995.85943446213	−2614628.95	0
**II** (*pS*-*pS*-*pR*)	−995.84829709311	−2614599.70	+29.24
**III** (*pR*-*pS*-*pR*)	−995.81984998676	−2614525.02	+103.93

4. Chirality transfer from enantiomeric 1 to 4 via trimerization

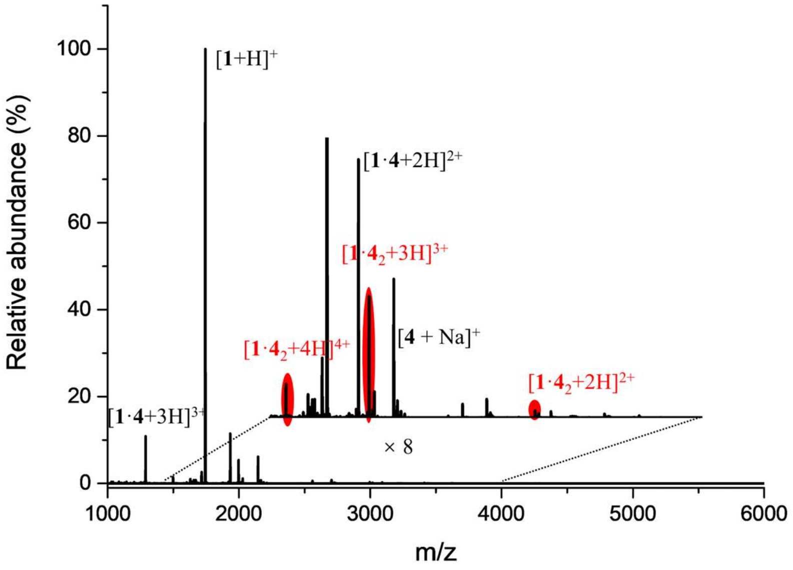

Supplementary Fig. 16 High resolution ESI-mass spectra of the mixture of *pS*-**1** and **4** (1:2). Signals corresponding to the ionized trimers are clearly observed along with those of monomeric and dimeric species, which might be formed during the process of ionization.

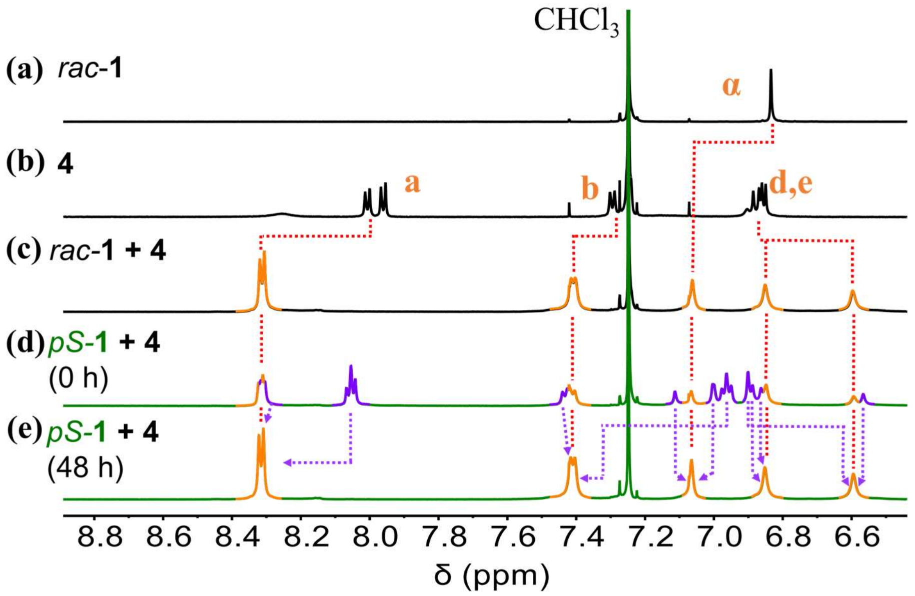

Supplementary Fig. 17 Partial ^{1}H NMR spectra (600 MHz, $CDCl_3$, 25 °C) of (a) racemic **1** (0.5 mM), (b) **4** (1.0 mM), (c) the mixture of racemic **1** (0.5 mM) and **4** (1.0 mM),[1] (d) the mixture of *pS*-**1** (0.5 mM) and **4** (1.0 mM) as prepared, and (e) the mixture of *pS*-**1** (0.5 mM) and **4** (1.0 mM) recorded after 48 h at room temperature. The as prepared mixture of *pS*-**1** and **4** possessed more than one species, while all species were finally re-assembled into the homochiral trimer within 48 h.

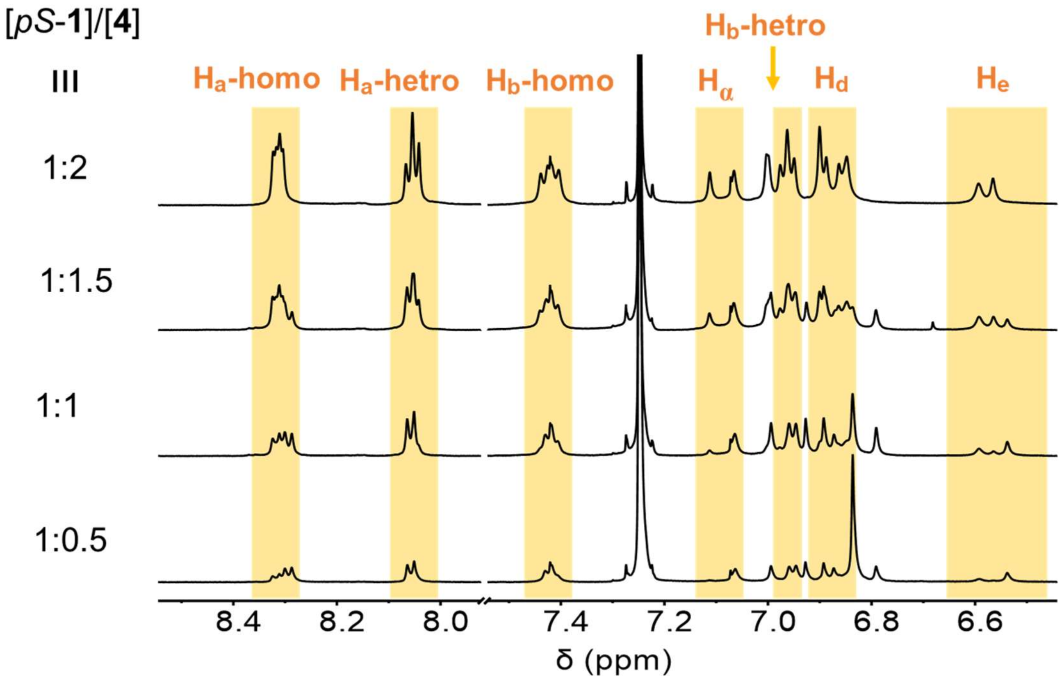

Supplementary Fig. 18 Partial ^{1}H NMR spectra (600 MHz, $CDCl_3$, 25 °C) of the mixtures of *pS*-**1** (0.5 mM) and **4** in various molar ratios. As the ratio was decreased, species of dimers were observed clearly.

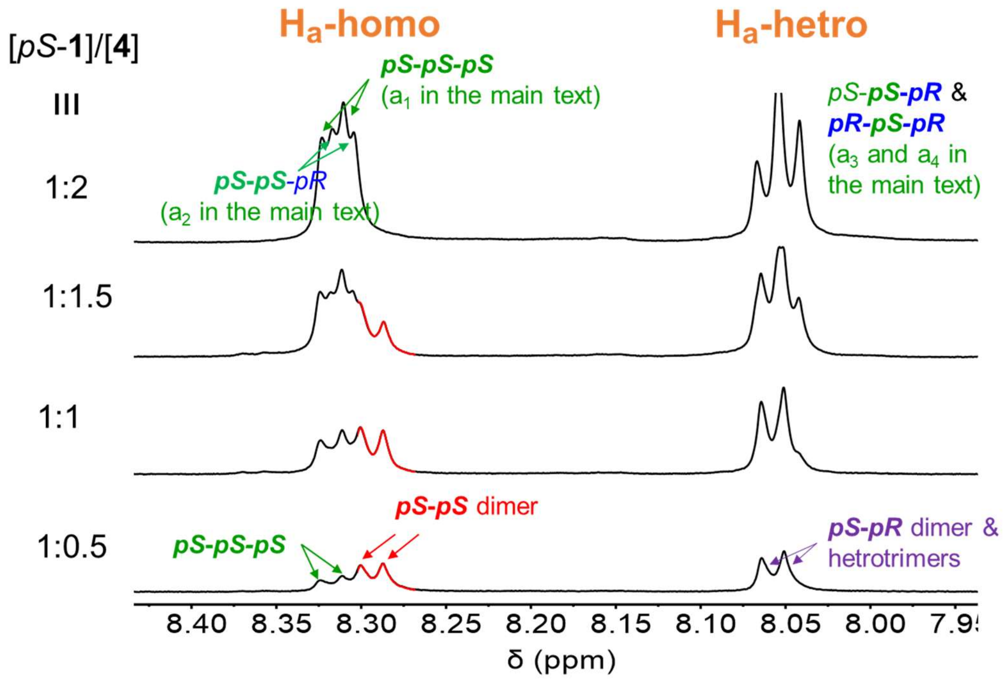

Supplementary Fig. 19 The signals of proton H_a (in the main text) on the ^{1}H NMR spectra (600 MHz, $CDCl_3$, 25 °C) of the mixtures of *pS*-**1** (0.5 mM) and **4** in various molar ratios. As the ratio was decreased, species of dimers were observed clearly.

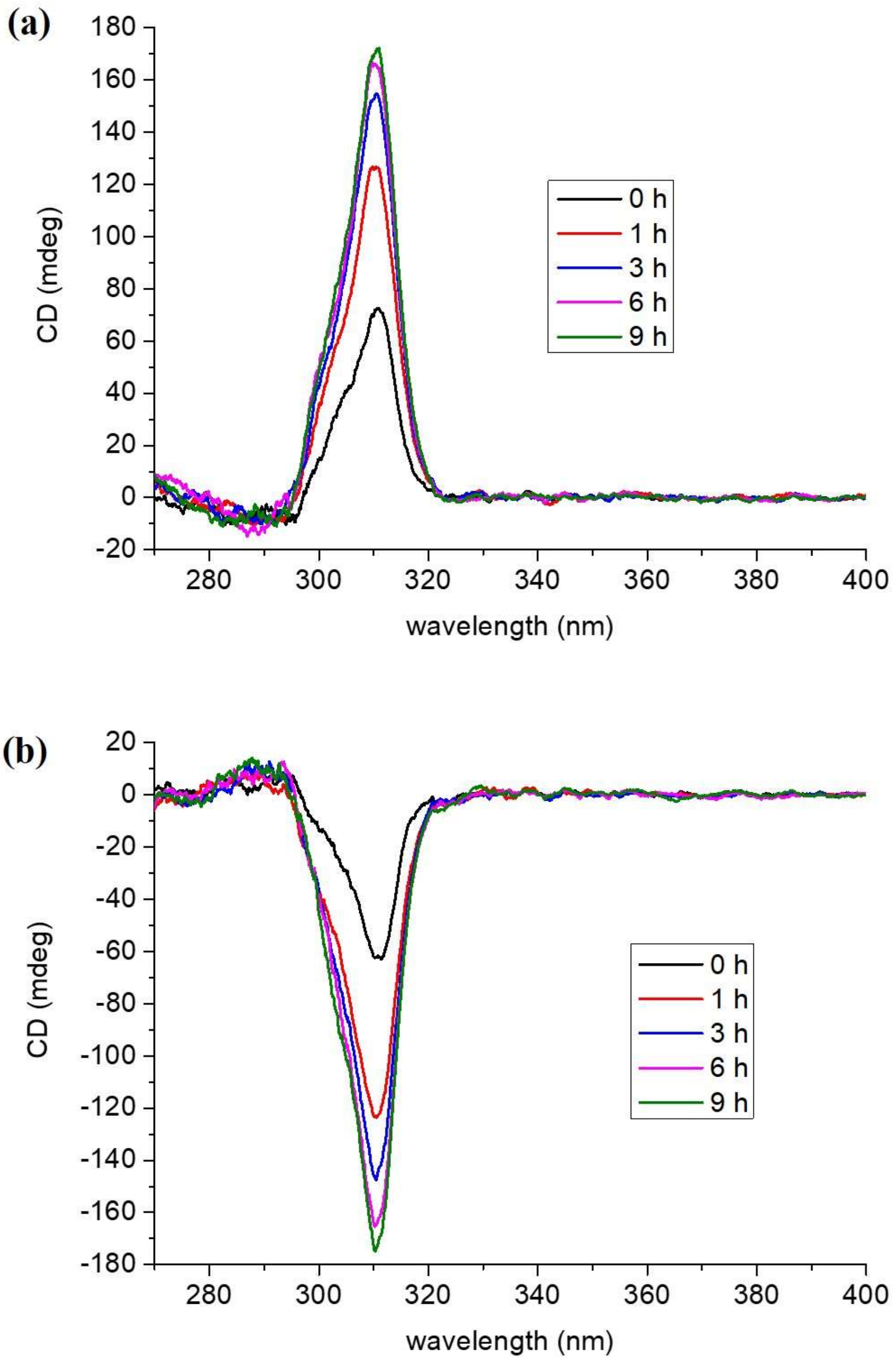

Supplementary Fig. 20 CD spectra of (a) the mixture of *pR*-**1** (0.5 mM) with **4** (1.0 mM) in chloroform upon heating at 50 °C and (b) the mixture of *pS*-**1** (0.5 mM) with **4** (1.0 mM) in chloroform upon heating at 50 °C.

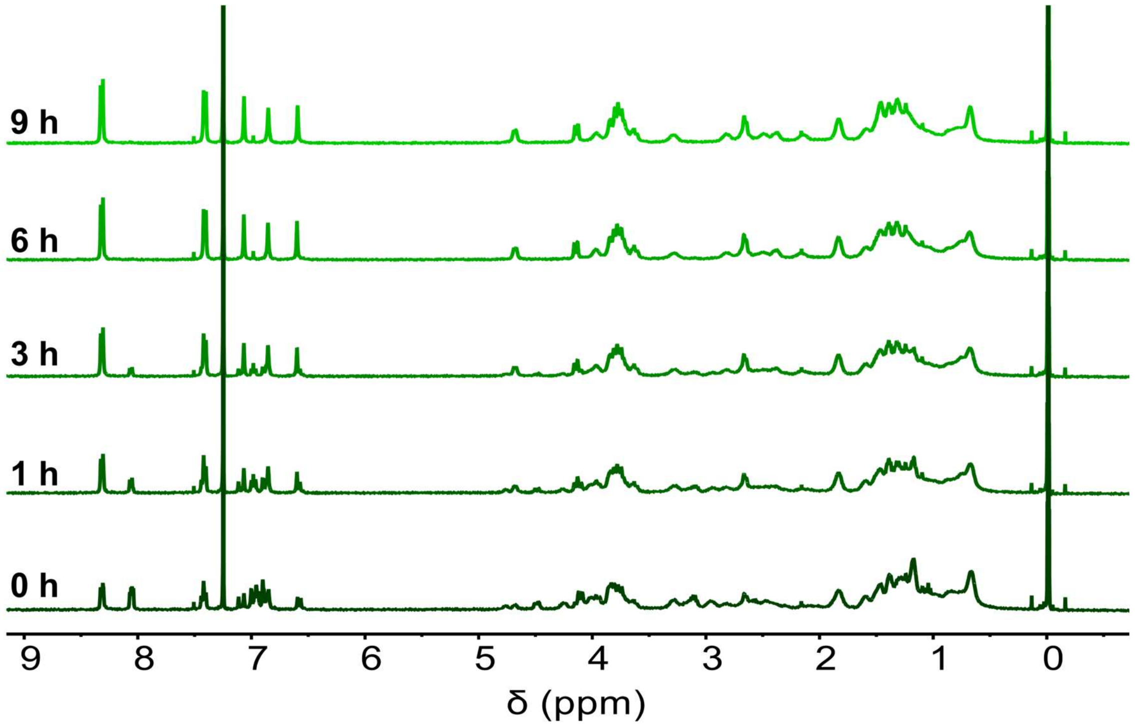

Supplementary Fig. 21 ^{1}H NMR spectra (400 MHz, $CDCl_3$) of the mixture of *pS*-**1** and **4** upon heating at 50 °C. The concentrations of **1** and **4** were 0.5 mM and 1.0 mM, respectively.

5. Chirality transfer from enantiomeric 1 to 3 via trimerization

As the rate of the chirality transfer process should be decreased by increasing the length of the alkyl chains of the acidic pillar[5]arene from 4 carbon atoms to 12, length tuning with the number of carbons between 4 and 12 would help to understand the conversion from trimer **III** to **II** and for that from **II** to **I**. Therefore, acidic pillar[5]arene **3** was synthesized, and the supramolecular assembly process of it with *pS*-**1** was investigated by ^{1}H NMR measurement. At 50 °C, the chirality transfer process was completed in 10 min (Supplementary Fig. 22), which is longer than that of the mixture of *pS*-**1** and **2**, but shorter than that of the mixture of *pS*-**1** with **4**.

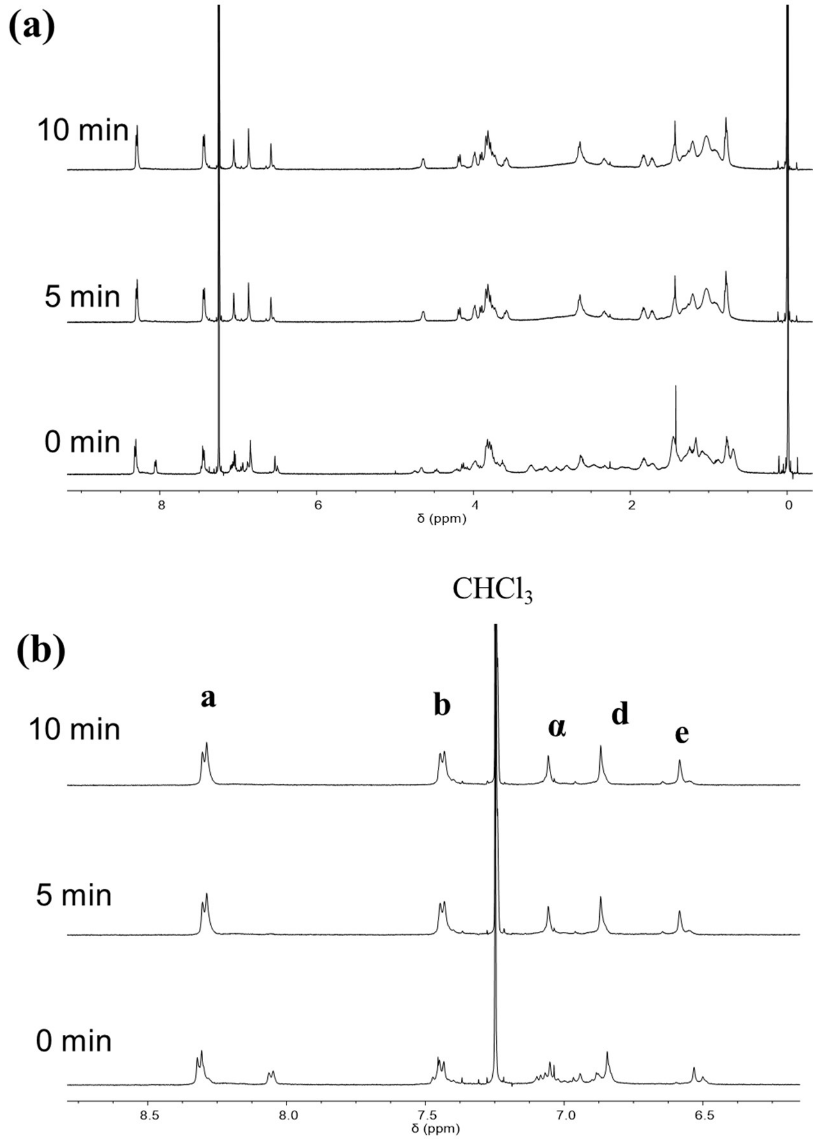

Supplementary Fig. 22 (a) Full and (b) partial ^{1}H NMR spectra (400 MHz, $CDCl_3$) of a mixture of *pS*-**1** and **3** upon heating at 50 °C. The concentrations of **1** and **3** were 0.5 and 1.0 mM, respectively.

6. Mechanism of the chirality transfer process

6.1 Determination of the rotation barriers of acidic pillar[5]arenes

Determination of the rotation barriers of pentaacid compounds **2**, **3** and **4** is of great help in understanding the mechanism of the difference in chirality transfer process of the mixture of **2**, **3** and **4** with enantiomeric **1**. Unfortunately, however, we cannot directly measure the inversion barriers of compounds **2**, **3** and **4** in chloroform. This is because the molecules contain carboxyl groups, which can act as both hydrogen bond acceptors and hydrogen bond donors, and the molecules will assemble in pairs through hydrogen bonds, which has been observed in our previous work.[1,8] To determine the rotation barriers of **2**, **3** and **4**, the analogs of **2**, **3** and **4**, that is, their unhydrolyzed ester containing precursors (namely, **ester-2**, **ester-3** and **ester-4**, respectively) were used. The volumes of the upper and lower rims of these analogs are similar to **2**, **3** and **4**, respectively.

The methylene of benzyl groups in molecules **ester-2**, **ester-3** and **ester-4** can split at lower temperatures and coalesce at higher temperatures on NMR spectra (Supplementary Figs. 23-25). Therefore, the free energy of rotaion $\Delta G^{\neq}$ can be calculated by using the following equation:[9] $\Delta G^{\neq} = 8.314T_{\mathrm{c}}(22.96 + \lg{(T_{\mathrm{c}}/\delta\nu)})$
where $\delta\nu$ is the chemical shift difference between the proton signals from the split methylene protons, and T_{c} is the coalescence temperature of these proton signals.

With the help of temperature variable ^{1}H NMR technology, their rotation barriers were determined to be 50.6, 56.2 and 59.4 kJ/mol, respectively, which are slightly lower than the corresponding pillar[5]arenes whose upper and lower rims are both alkyl groups of the same length.[10] We believe that the five bulky benzoate groups at one rim of the molecule may make the opening sizes of the two rims of the pillar[5]arenes **ester-2**, **ester-3** and **ester-4** different. The molecule is folded towards the rim of alkyl chains, thus making it easier for the molecule to rotate through the alkyl side.

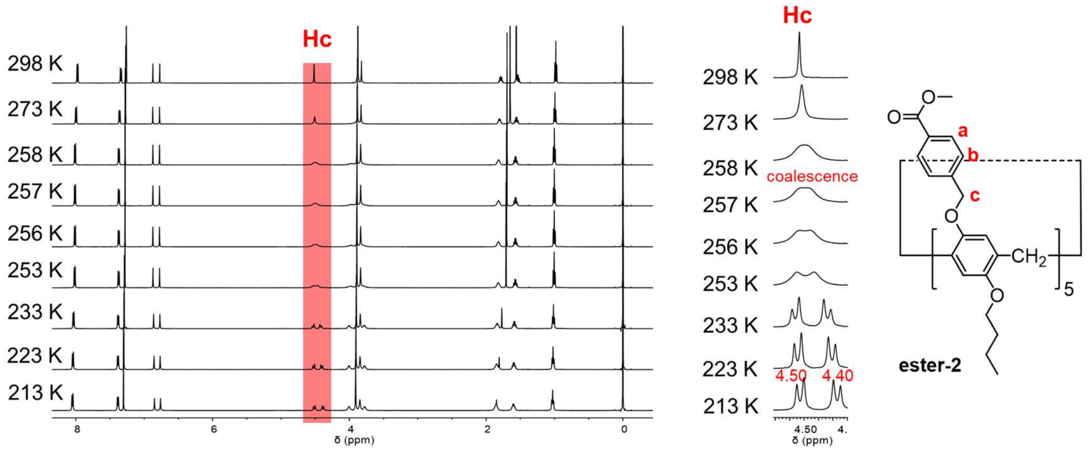

Supplementary Fig. 23 Full and partial ^{1}H NMR spectra (400 MHz, $CDCl_3$) at various temperatures and the molecular structure of **ester-2**. The rotation barrier was determined to be 50.6 kJ/mol.

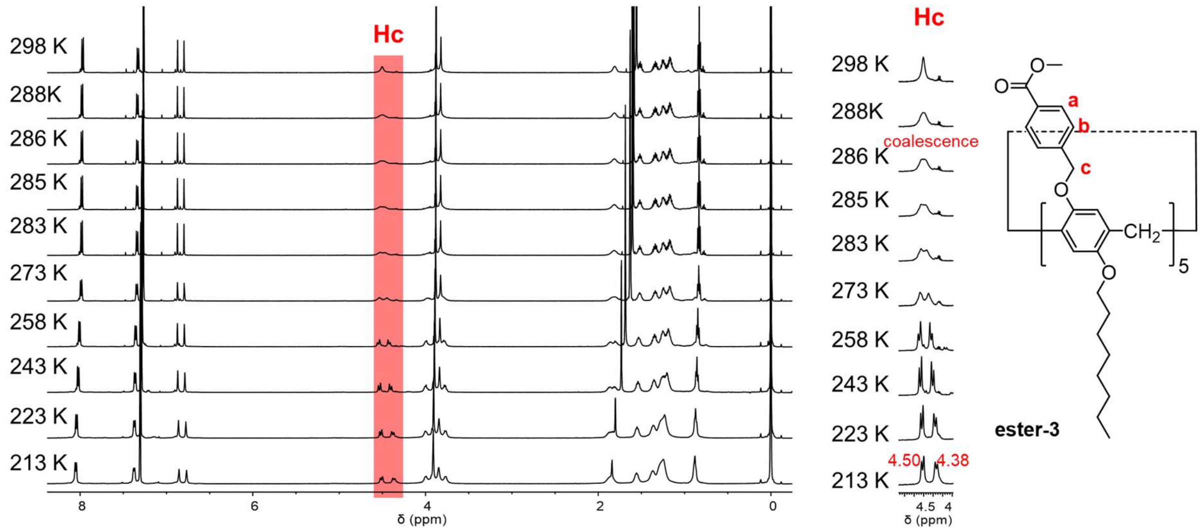

Supplementary Fig. 24 Full and partial ^{1}H NMR spectra (400 MHz, $CDCl_3$) at various temperatures and the molecular structure of **ester-3**. The rotation barrier was determined to be 56.2 kJ/mol.

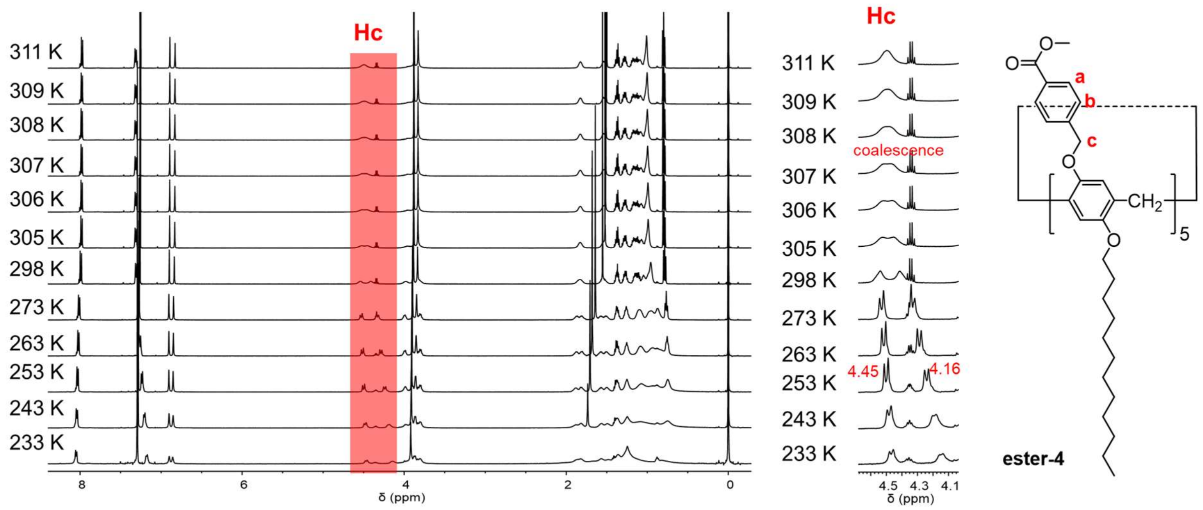

Supplementary Fig. 25 Full and partial ^{1}H NMR spectra (400 MHz, $CDCl_3$) at various temperatures and the molecular structure of **ester-4**. The signals of H_c broaden and are difficult to determine the accurate chemical shift at temperatures lower than 253 K. Therefore, the δν value was used that of 253 K. The rotation barrier was determined to be 59.4 kJ/mol.

6.2 Discussion on the mechanism of the chirality transfer process

Considering that there is no significant difference in the single molecule rotation barriers (more precisely the rotation barriers of their unhydrolyzed ester precursors) for compounds **2**, **3** and **4**, The chirality transfer rate of the 2:1 mixture of these molecules with enantiomeric **1** did range from 0 seconds to 9 h (at 50 °C). This makes a complete understanding of the mechanism of the chirality transfer process in this system very difficult. We propose the following possibilities:

1. The rotation barriers of their unhydrolyzed ester precursors of compounds **2**, **3** and **4**

do not really reflect their own rotation barriers. The molecules of **2**, **3** and **4** in chloroform could interact with each other through five pairs of hydrogen bonds, producing dimers. As the length of alkyl chains on one rim of the pillar[5]arene increasing, the steric hindrance of that rim increases consequently. This may cause the five benzoic acid groups on the other rim more compact, so that the strength of its intermolecular or intramolecular hydrogen bond network will be enhanced, and then the rotation barrier of the acidic pillar[5]arene will be raised.

2. Due to the large steric hindrance of the five benzoic acid groups, the acidic pillar[5]arenes **2**, **3** and **4** may not be perfectly pillar-shaped, so the longer the alkyl chain on the opposite rim may make the entire pillar[5]arene molecule closer to the pillar-shape, thereby adding additional stability to the trimeric nanotubes formed after mixing with enantiomeric **1**, making the assemblies less prone to dissociation.

3. Although the alkyl chains are not the ideal guests of the cavity of pillar[5]arenes, they can still be recognized by pillar[5]arenes to some extent. Thus, longer alkyl chains can prevent the rotation of the acidic pillar[5]arenes by either entering its own cavity or inserting into the cavity of other pillar[5]arenes, and even make the acidic pillar[5]arenes less likely to dissociate from their trimeric nanotubular assemblies.

7. Solvent effect on the chirality transfer

Solvents have significant effects on many supramolecular systems. Due to solubility and assembly issues, we further chose dichloroethane (DCE) and tetrachloroethane (TCE), and tetrahydrofuran (THF) as solvents to investigate the solvent effect on the chirality transfer process. In order to increase the solubility, we added 1% chloroform to DCE and TCE. In summary, we investigated three more solvent systems in addition to chloroform, namely DCE + 1% $CHCl_3$, TCE + 1% $CHCl_3$ and THF.

We selected the 1:2 mixture of *pS*-**1** and **2** as the representative system. This is mainly due to the fast chirality transfer of the mixture of *pS*-**1** and **2** in chloroform. Changing the solvent for this system may slow down the chirality transfer process described above.

We found that the chirality transfer was completed after 1 h in the solvent mixture of DCE + 1% $CHCl_3$, while it finished immediately after the mixing in the other two solvent systems, which is alike the observation in chloroform (Supplementary Figs. 26-28). It is probable that DCE molecules can enter the cavity of pillar[5]arene **2** as guests, thereby reducing the rate at which it is induced by the chirality of **1**.[4] However, THF and TCE are bulky like chloroform and cannot serve as guest molecules of pillar[5]arenes, so the chirality transfer in them is as fast as in chloroform.

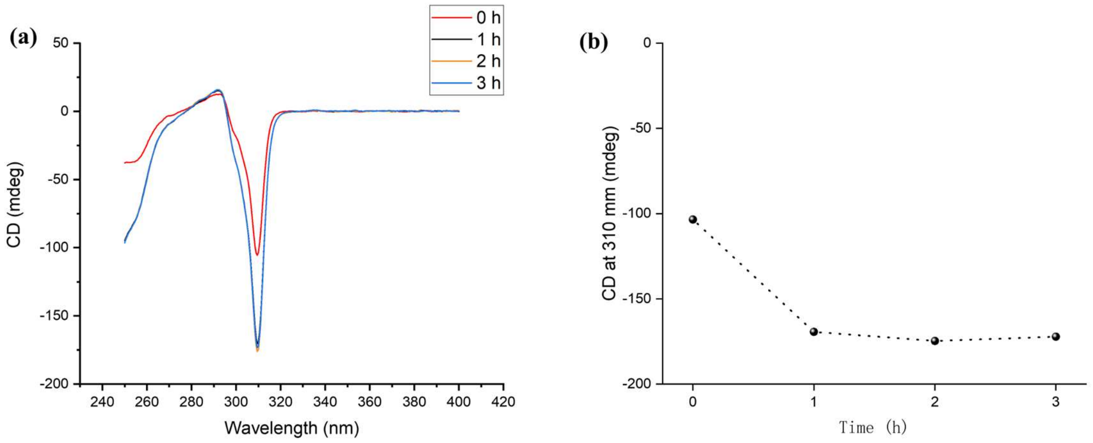

Supplementary Fig. 26 (a) Full CD spectra and (b) CD intensity at 310 nm of the mixture of **2** with *pS*-**1** in DCE (containing 1% of chloroform) at room temperature. The concentrations of **1** and **2** were 0.5 mM and 1.0 mM, respectively.

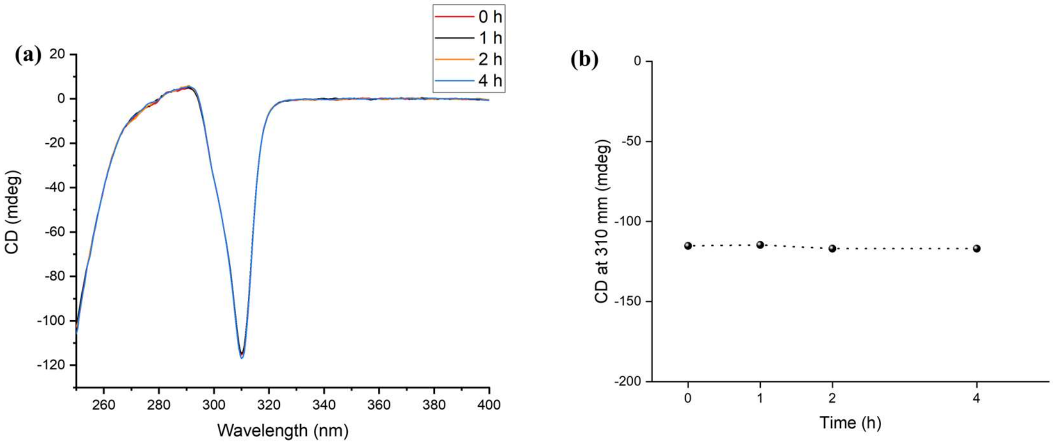

Supplementary Fig. 27 (a) Full CD spectra and (b) CD intensity at 310 nm of the mixture of **2** with *pS*-**1** in TCE (containing 1% of chloroform) at room temperature. The concentrations of **1** and **2** were 0.5 mM and 1.0 mM, respectively.

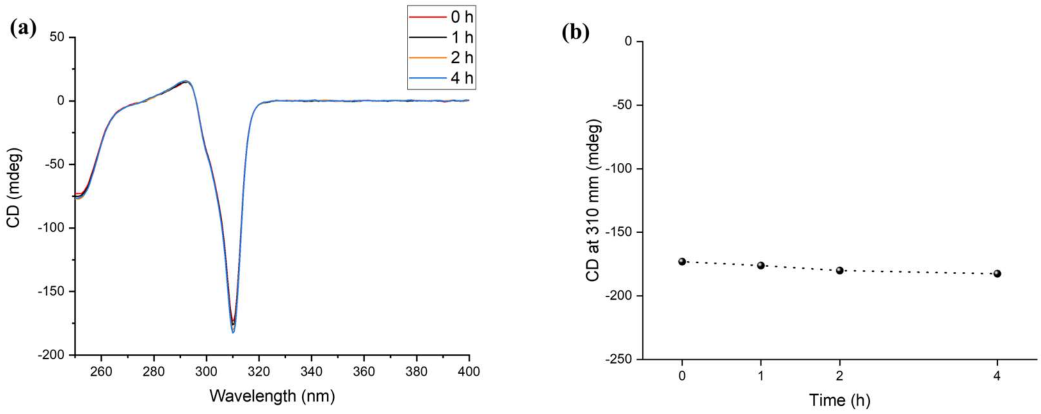

Supplementary Fig. 28 (a) Full CD spectra and (b) CD intensity at 310 nm of the mixture of **2** with *pS*-**1** in THF at room temperature. The concentrations of **1** and **2** were 0.5 mM and 1.0 mM, respectively.

Supplementary Note 1

It is difficult for us to determine the barriers of the two transformations, because the reaction rate of the chirality transfer process became difficult to precisely control as the temperature decreased. For instance, at 23 °C, the whole process was completed within 48 h once (Supplementary Fig.29), while it was much faster in another measurement. However, it could not complete within 72 h sometimes. We speculated that some unknown factors influenced the chirality transfer process together with the effect of temperature. Factors like water content, shaking of the system and the sequence of mixing were gone through. However, we have no confidence in claiming how these factors affect the system at this stage. In contrast, at a higher temperature (e.g., 50 °C), the rate of the chirality transfer was much easier to control. We believe that heating accelerated the reaction, which made temperature the decisive factor, while the influence of other factors was so small that may be ignored. Related research is being carried out in-depth in our group now.

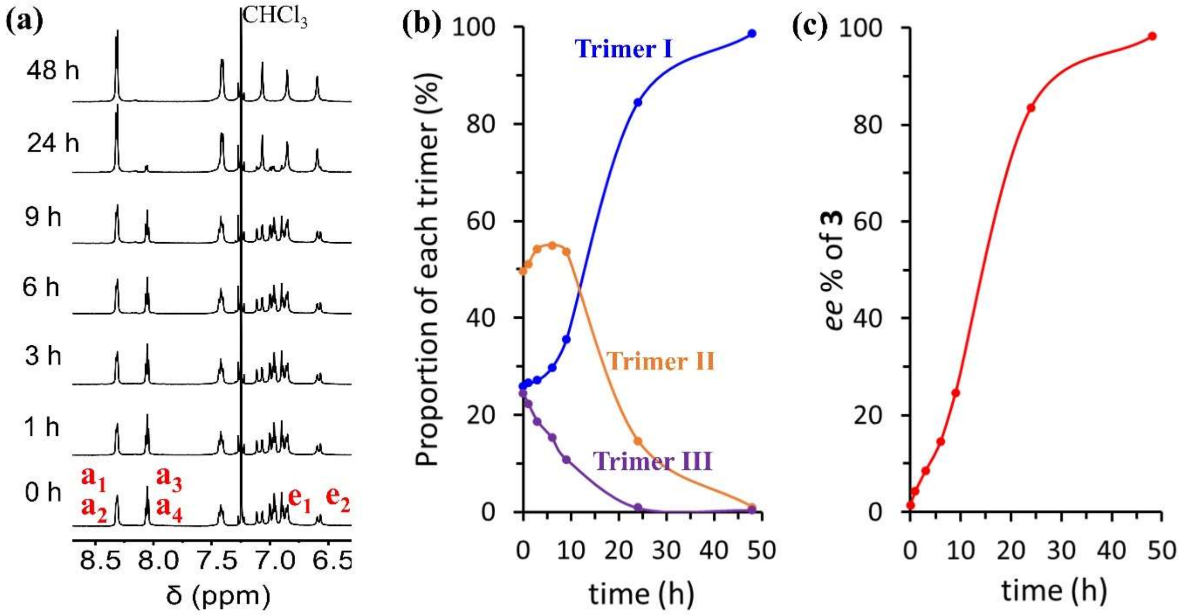

Supplementary Fig. 29 One of the examples of chirality transfer of the mixture of *pS*-**1** (0.5 mM) and **4** (1.0 mM) at room temperature. (a) Partial ^{1}H NMR spectra (600 MHz, $CDCl_3$). (b) Distribution of the three trimers. (c) Chiral induction of **4**.

Supplementary References

1. Fa, S., Sakata, Y., Akine, S. & Ogoshi, T. Non-covalent interactions enable the length-controlled generation of discrete tubes capable of guest exchange. *Angew. Chem. Int. Ed.* **59,** 9309–9313 (2020).
2. Strilets, D., Fa, S., Hardiagon, A., Baaden, M., Ogoshi, T. & Barboiu, M. Biomimetic approach for highly selective artificial water channels based on tubular pillar[5]arene dimers. *Angew. Chem. Int. Ed*. **59**, 23213–23219 (2020).
3. Ogoshi, T., Ueshima, N., Yamagishi, T., Toyota Y. & Matsumi, N. Ionic liquid pillar[5]arene: its ionic conductivity and solvent-free complexation with a guest. *Chem. Commun.* **48,** 3536–3538 (2012).
4. Nagata, Y., Suzuki, M., Shimada, Y., Sengoku, H., Nishida, S., Kakuta, T., Yamagishi, T., Suginome, M. & Ogoshi, T. Holding of planar chirality of pillar[5]arene by kinetic trapping using host–guest interactions with achiral guest solvents. *Chem. Commun.* **56,** 8424–8427 (2020).
5. Neese, F., Wennmohs, F., Becker, U. & Riplinger, C. The ORCA quantum chemistry program package. *J. Chem. Phys.* **152**, 224108 (2020).
6. Bannwarth, C., Ehlert, S. & Grimme, S. GFN2-xTB-An accurate and broadly parametrized self-consistent tight-binding quantum chemical method with multipole electrostatics and density-dependent dispersion contributions. *J. Chem. Theory Comput.* **15**, 1652–1671 (2019).
7. Marenich, A. V., Cramer, C. J. & Truhlar, D. G. Universal solvation model based on solute electron density and on a continuum model of the solvent defined by the bulk dielectric constant and atomic surface tensions. *J. Phys. Chem. B* **113**, 6378–6396 (2009).
8. Fa, S., Adachi, K., Nagata, Y., Egami, K., Kato, K. & Ogoshi, T. Pre-regulation of the planar chirality of pillar[5]arenes for preparing discrete chiral nanotubes. *Chem. Sci.* **12,** 3483–3488 (2021).
9. Ogoshi, T., Yamafuji, D., Aoki, T. & Yamagishi, T. Photoreversible transformation between seconds and hours time-scales: threading of pillar[5]arene onto the azobenzene-end of a viologen derivative. *J. Org. Chem.* **76,** 9497–9503 (2011).
10. Ogoshi, T., Kitajima, K., Aoki, T, Fujinami, S., Yamagishi, T. & Nakamoto, Y. Synthesis and conformational characteristics of alkyl-substituted pillar[5]arenes. *J. Org. Chem.* **75,** 3268–3273 (2010).
