# Supplementary material for: Real-time chirality transfer monitoring from statistically random to discrete homochiral nanotubes
Source: Nat Commun. 2022 Nov 30;13:7378. doi: 10.1038/s41467-022-34827-z (PMC9712533; doi:10.1038/s41467-022-34827-z)
Supplement: Supplementary file 3 — Description of Additional Supplementary Files [file 41467_2022_34827_MOESM3_ESM.pdf]

Description of Additional Supplementary Files

Supplementary Data 1: Optimized structures of the homochiral and heterochiral trimers in xyz format.
